# TNF superfamily molecules in atherosclerosis: mechanistic insights and therapeutic translation

**DOI:** 10.3389/fimmu.2025.1629577

**Published:** 2025-11-05

**Authors:** Jia Luo, Jiaying Zhang, Yunfei Xie, Mengya Wu, Zitong Wang, Di Ma

**Affiliations:** ^1^ Bethune First Clinical School of Medicine, The First Hospital of Jilin University, Changchun, China; ^2^ Department of Neurology and Neuroscience Center, The First Hospital of Jilin University, Changchun, Jilin, China

**Keywords:** atherosclerosis, TNF superfamily, co-stimulatory molecules, immune checkpoints, inflammation

## Abstract

Atherosclerosis (AS) is the core pathological mechanism underlying myocardial infarction and stroke, which are among the leading causes of death worldwide. The landmark CANTOS trial provided robust validation of anti-inflammatory immunotherapy as a viable approach for AS treatment, thereby underscoring the critical role of inflammatory-immune dysregulation in the pathogenesis of AS. Members of the tumor necrosis factor superfamily (TNFSF), acting as key co-stimulatory immune checkpoints, exhibit spatiotemporally precise regulatory effects on the progression of AS. They achieve this by modulating lipid metabolic disorders, dynamic plaque evolution, and thrombotic complications. Numerous TNFSF-targeted immunotherapeutic have been introduced into clinical practice, showing significant efficacy in oncology and autoimmune diseases. This offers novel insights into the dissection of the TNFSF immune network and the development of therapeutic targets. This review aims to systematically analyze the mechanistic roles of TNFSF co-stimulatory molecules in AS pathology. It also synthesizes current clinical trial outcomes and approved drug profiles, emphasizing their great potential as biomarkers and therapeutic targets for AS and related cardiovascular diseases. Furthermore, it outlines future directions in drug discovery, highlighting that targeting TNFSF downstream signaling pathways and cell type-specific therapies may emerge as groundbreaking strategies for effective AS management.

## 1 Introduction

Atherosclerosis is the core pathological basis of cardiovascular diseases, characterized by chronic inflammatory responses in the vascular wall. Currently, statins, which lower low-density lipoprotein cholesterol levels, remain the cornerstone of AS treatment. However, some patients exhibit poor drug tolerance or residual inflammatory risk, indicating that lipid-lowering strategies alone are insufficient to fully control disease progression and cannot completely block inflammation-driven plaque advancement (1). The CANTOS trial first demonstrated that anti-inflammatory therapy could reduce cardiovascular event rates independently of lipid-lowering effects, marking the transformation of the “inflammatory hypothesis” of atherosclerosis into a clinically actionable “inflammatory theory”. This discovery has spurred the development of precision therapeutic strategies targeting inflammatory pathways (2). Drugs such as NLRP3 inhibitors, colchicine and IL-6 inhibitors have shown clinical potential in treating atherosclerotic cardiovascular disease (ASCVD) (3–5). However, broad-spectrum anti-inflammatory strategies may lead to immunosuppression-related side effects (e.g., increased infection risk), necessitating the exploration of more targeted immune modulation pathways.

In recent years, the role of immune checkpoint regulation in AS has garnered significant attention. Numerous preclinical studies have demonstrated that targeting immune checkpoints can inhibit AS progression and enhance plaque stability. Drobni et al. (6) found that PD-1/PD-L1 inhibitor therapy increased the risk of atherosclerotic cardiovascular events by 4.8-fold, providing clinical evidence for the role of immune checkpoints in AS. Notably, members of the tumor necrosis factor superfamily (TNFSF) not only act as classical pro-inflammatory factors but also finely regulate T-cell activation through costimulatory molecules such as OX40/OX40L, 4-1BB/4-1BBL, and CD40/CD40L, thereby influencing innate and adaptive immune responses. The dual roles of these molecules (pro-inflammatory and immunomodulatory) in AS make them potential targets for precision intervention.

While the association between immunoglobulin superfamily checkpoint molecules, such as PD-1 and PD-L1, and AS has been extensively reviewed, systematic summaries of costimulatory molecules within the TNFSF remain lacking. Existing studies often focus on single molecules, such as tumor necrosis factor-alpha (TNF-α), while overlooking the synergistic or antagonistic networks among TNFSF members. Furthermore, innovative therapies targeting TNFSF costimulatory pathways, such as CD40 antagonists, which have been successfully applied in autoimmune diseases, offer cross-disciplinary insights for AS treatment. However, their translational potential in cardiovascular medicine has yet to be fully explored. This review elucidates the mechanisms of costimulatory checkpoints within the TNFSF in atherosclerosis and their clinical research progress, while evaluating innovative strategies targeting TNF costimulatory molecules, aiming to provide references for further research and clinical applications in this field.

## 2 The pathogenesis of atherosclerosis in the context of immunology

### 2.1 The pathological process of atherosclerosis

**Figure 1:**
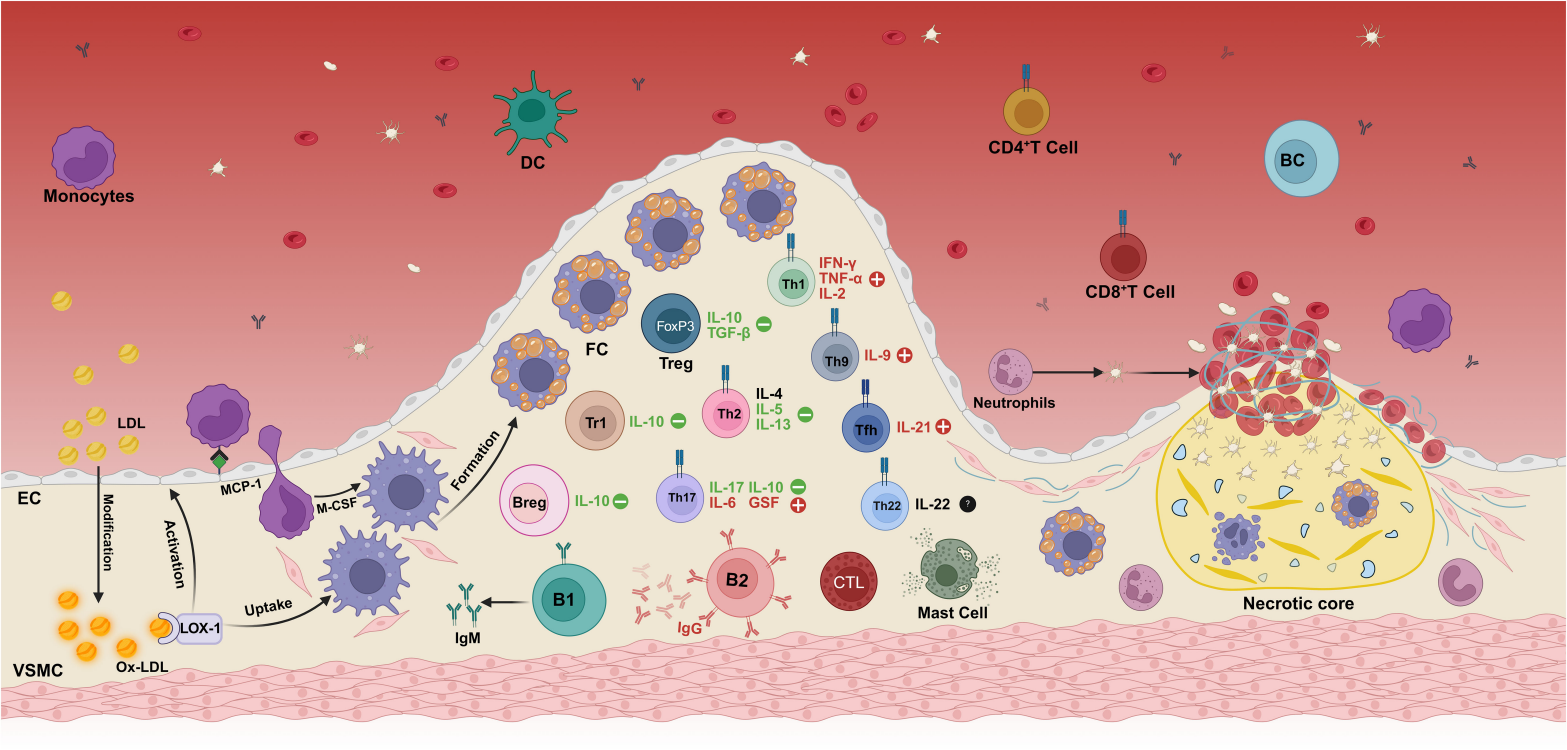
Immunopathogenesis mechanism of atherosclerosis. The deposition of modified oxidized low-density lipoprotein (oxLDL) in the intima of the vascular wall is the initial step in the development of atherosclerosis. In addition to scavenger receptors, oxLDL also binds to lectin-like oxidized low-density lipoprotein receptor-1 (LOX-1). The binding of oxLDL to LOX-1 initiates a diverse array of cellular processes across different cell types. Specifically, it significantly enhances the uptake of oxLDL in both macrophages and vascular smooth muscle cells (VSMCs), thereby promoting the formation of foam cells. Moreover, this interaction also plays a crucial role in endothelial activation (LOX-1 is the identical oxLDL receptor expressed on endothelial cells, macrophages, and VSMCs). This process promotes innate immune cells, such as monocytes and neutrophils, to infiltrate into the intima of blood vessels, thereby aggravating the formation and development of atherosclerotic plaques. Infiltrating monocytes differentiate into macrophages under the action of the local microenvironment and transform into foam cells by massive uptake of oxLDL, which constitutes the main cellular component of the plaque. With the progression of the disease, adaptive immune cells—including effector T cell subsets (Th1, Th9, Th17), regulatory T cells (Treg), and B cells are successively recruited to the lesion site, and play a complex regulatory role in the pathophysiological process of atherosclerosis by secreting a variety of pro-inflammatory or anti-inflammatory cytokines. During plaque evolution, the accumulation of foam cells, cellular debris, and cholesterol forms a necrotic core within the plaque, further exacerbating the local inflammatory response. In the terminal stage of the disease, unstable plaques rupture, exposing the collagen fibers under the intima, prompting the release of von Willebrand factor and activation of platelets, ultimately leading to thrombosis. Created in https://BioRender.com.

Atherosclerosis is a long-term, lipid-driven, immune-mediated autoinflammatory disease that is considered to be a potentially serious immunopathological event. Its pathogenesis is complex, covering multiple stages such as endothelial dysfunction, fatty streak formation, fibrous plaque formation and plaque rupture (**Figure 1**).

In the physiological state, the vascular endothelium is composed of endothelial cells (ECs) that form a heterogeneous monolayer facing the luminal side of all blood vessels and serve as the first barrier to circulating components in the bloodstream (7). However, damaging factors such as free radicals, lipid accumulation, and abnormal blood flow can lead to endothelial cell activation, endothelial dysfunction, and increased vascular permeability. Low-density lipoprotein (LDL) deposited in the intima of blood vessels is oxidized to oxidized low-density lipoprotein (oxLDL), which in turn induces the activation of endothelial cells and vascular smooth muscle cells. This activation enhances nuclear factor kappa-β (NF-κB) production within ECs, leading to the upregulation of leukocyte adhesion molecules and chemokines. These mediators promote the rolling of monocytes and neutrophils on the vascular surface and their subsequent adhesion to the activated endothelium (8, 9). Monocyte chemoattractant protein-1 (MCP-1) further induces adherent monocytes to enter the tunica intima and mature into macrophages upon colony-stimulating factor (CSF) stimulation (10). Macrophages up-regulate pattern recognition receptors, including Toll-like receptors (TLRs) and scavenger receptors (SRs). Activation of TLR pathways leads to inflammatory responses, while SRs mediate the uptake of oxLDL particles and the subsequent formation of foam cells, which aggregate to constitute the earliest atherosclerotic lesions-lipid streaks (9). With the development of atherosclerosis, macrophages, vascular smooth muscle cells and foam cells in the plaque undergo apoptosis and necrosis, forming a necrotic core (11).

### 2.2 Role of immune cells in atherosclerosis

CD4^+^ T cells are central orchestrators of adaptive immunity and can differentiate into a spectrum of helper T (Th) or regulatory T (Treg) cell subsets. Following antigen presentation by antigen-presenting cells (APCs), lesion-infiltrating CD4^+^ T cells give rise to distinct Th subtypes, including Th1, Th2, Th9, Th17, Th22, T follicular helper (Tfh) cells and CD28-negative T cells, as well as to Treg subsets such as forkhead box P3-positive (FOXP3^+^) Treg cells and type 1 regulatory T (Tr1) cells (12, 13). Th1 cells secrete the pro-inflammatory cytokines interleukin-2 (IL-2), interferon-γ (IFN-γ) and TNF-α, which initiate or amplify atherosclerotic inflammation by activating monocytes, macrophages and dendritic cells (DCs) and by undermining the stability of Treg cells, thereby enhancing plaque vulnerability (14). Th2 cells secrete IL-4, IL-5 and IL-13, among which IL-5 and IL-13 have been shown to have a protective effect on atherosclerosis, and IL-13 can reduce the infiltration of macrophages in plaques by decreasing the expression of vascular cell adhesion molecule-1 (VCAM-1) (12). However, the role of IL-4 remains unclear. Th9 cells mainly secrete IL-9, and transforming growth factor-beta (TGF-β) and IL-4 stimulate Th9 cells to produce IL-9 (13). Some studies have suggested that IL-9 may mediate the infiltration of inflammatory cells into atherosclerotic lesions by inducing the expression of VCAM-1 in aortic endothelial cells, thereby promoting atherosclerosis (14). Th17 cells can express either pro-atherogenic (IL-6, granulocyte stimulating factor, chemokines) or anti-atherogenic (IL-17, IL-10) inflammatory molecules in different environments (12). Th22 cells express the transcription factor aryl hydrocarbon receptor and produce IL-22. The role of Th22 is still unclear. IL-22 may be involved in the activation of vascular repair by stimulating the differentiation of medial vascular smooth muscle cells (VSMCs) into a synthetic phenotype, promoting the migration of VSMCS into the intima and causing plaque growth (15). However, it has also been suggested that Th22 can reduce atherosclerosis by inhibiting gut microbiota (16). Treg cells are a subset of T cells that control the autoimmune response *in vivo*, and they inhibit the inflammatory response within the plaque to exert a protective role. Treg protects against atherosclerosis by secreting IL-10 and TGF-β, among which IL-10 is an anti-inflammatory factor and TGF-β can stabilize plaque formation (17, 18). Tr1 plays an inhibitory role in atherosclerosis mainly by secreting IL-10 (19). In addition to CD4^+^ T cells, cytotoxic CD8^+^ T cells are also found in atherosclerotic lesions. CD8^+^ T cells can induce apoptosis of target cells by inducing the release of cytotoxins, perforin and granzyme. Once activated, CD8^+^T cells can also produce large amounts of pro-inflammatory cytokine IFN-γ, thereby promoting the formation of atherosclerosis (20). The activation of both CD4^+^ and CD8^+^ T cells is controlled by immune checkpoint proteins and can occur in secondary lymphoid organs, local atherosclerotic lesions, or possibly arterial tertiary lymphoid organs (21).

Although B cells are relatively scarce in atherosclerotic plaques compared to other immune cells, Breg cells play different roles through their various subtypes, such as B1 and B2. B1 cells exhibit potent anti-atherogenic effects by secreting IgM antibodies that recognize the surface antigenic determinants of apoptotic cells and oxLDL, thereby inhibiting foam cell formation and blocking oxLDL uptake (22, 23). Similarly, Breg cells can inhibit the activation of Th cells by secreting IL-10, reducing antigen presentation by macrophages and the production of pro-inflammatory cytokines. Together with B1 cells, Breg cells form a protective axis that stabilizes the plaque by suppressing the inflammatory response. However, most studies seem to suggest that B2 cells have a pro-inflammatory role, which promotes the development of atherosclerosis through the production of IgG antibodies, the activation of T cells and the secretion of pro-inflammatory factors (such as IFN-γ) (24).

Other immune cells also play an important role in the pathogenesis of atherosclerosis. Macrophages in plaques are mainly divided into M1 and M2 types, and their differentiation is stimulated by local oxLDL and other stimuli, which play different roles in atherosclerosis (25). M1 macrophages secrete the pro-inflammatory cytokines IL-1β, IL-6 and TNF-α, and also produce the chemokines C-X-C motif chemokine ligand 9 (CXCL9) and CXCL10, thereby recruiting immune cells, amplifying inflammation, promoting lesion expansion and destabilizing plaques (29). M2 macrophages have anti-inflammatory effects, promote lipid clearance and anti-inflammatory factor (IL-10) secretion, inhibit the persistent recruitment of immune cells by eliminating local apoptotic cells, and contribute to tissue repair and inflammation resolution (26). The balance of M1/M2 is a dynamic process and is considered to be an important driver of plaque formation, progression and vulnerability. In addition, macrophages and DCs within the plaque can also act as APCs to further promote inflammatory responses by activating and recruiting T cells (27). Neutrophils mainly play a role after plaque rupture. They interact with activated platelets through the P-selectin signaling mechanism to form neutrophil extracellular traps and accelerate thrombosis (28, 29). Mast cells also play an important role in the evolution of atherosclerotic plaques. The various enzymes and cytokines released by them can degrade the extracellular matrix and induce further infiltration of inflammatory cells, thereby enhancing the local inflammatory response, which in turn leads to the progression and instability of the plaque (30).

## 3 Mechanisms of action of the TNF superfamily

**Figure 2:**
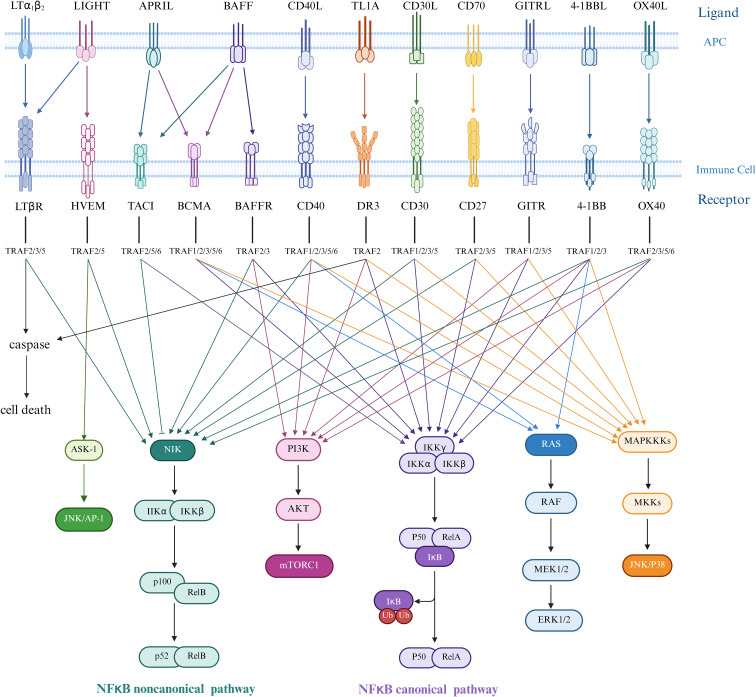
Ligand-receptor interactions in the TNF/TNFR superfamily and TRAF-mediated downstream signaling pathways. Ligands of the tumor necrosis factor superfamily (TNFSF) in trimeric form bind to receptors expressed as transmembrane proteins on two distinct but interacting cells—the upper representing the antigen-presenting cell (APC) membrane and the lower the immune cell membrane. The tumor necrosis factor receptor superfamily (TNFRSF) engages various TNF receptor-associated factor (TRAF) proteins to initiate downstream signaling cascades. In canonical NF-κB signaling, upstream signals recruit the IKK complex (comprising IKKα, IKKβ, and regulatory subunit IKKγ/NEMO). Activated IKKα and IKKβ phosphorylate the inhibitory protein IκBα, triggering its ubiquitination and proteasomal degradation. This releases the p50/RelA heterodimer, which translocates to the nucleus to function as a transcription factor, inducing target gene expression, predominantly regulating cell survival, inflammatory responses, and immune activation. In contrast, non-canonical NF-κB signaling is initiated by receptors such as CD40, RANK, LTβR, and BAFF-R, which activate NF-κB-inducing kinase (NIK). NIK subsequently phosphorylates and activates IKKα, mediating proteolytic processing of p100 into p52. The resultant p52/RelB complex translocates to the nucleus to regulate genes critical for lymphocyte development, survival, maturation, and adhesion.ERK1/2 promotes smooth muscle cell (SMC) migration and fibrous cap thickening, while JNK/p38 drives inflammatory cytokine and matrix metalloproteinase (MMP) expression, leading to plaque degradation and macrophage apoptosis. AP-1 integrates signals from multiple pathways to amplify inflammatory responses. mTORC1 suppresses autophagy, increases reactive oxygen species (ROS) production, and enhances lipid synthesis in both SMCs and macrophages, thereby promoting foam cell formation. The NF-κB transcription factor critically mediates inflammatory responses and cell death mechanisms in atherosclerosis pathogenesis. Created in https://BioRender.com.

The TNFSF comprises a complex signaling regulatory system involving ligands (TNFSF) and their receptors (TNFRSF), currently known to include 19 ligands and 29 receptors (**Figure 2**). Their specific interactions mediate cell survival, apoptosis, differentiation, and inflammatory responses (31).

TNF ligands bind to cell surface receptors in trimeric forms, mediating distinct signaling mechanisms through three types of receptors: (1) Death receptors contain an intracellular death domain (DD) and induce apoptosis by activating caspase cascades (32, 33); (2) TRAF-binding receptors lack a DD but possess a TRAF-interacting motif (TIM), activating downstream pathways by recruiting TNF-receptor-associated factor (TRAF) proteins (34). Notably, the TRAF protein family includes six canonical members (TRAF1-6) and one non-canonical member, TRAF7, which features a WD40 domain and serves as a hub in signal transduction (**Figure 2**) (35); (3) Type III receptors function as decoy receptors due to the absence of intracellular domains and lack of signal transduction capabilities (36, 37).

Death receptor signaling pathway: Taking TNFR1 as an example, TNF-α binding recruits TNF receptor-associated death domain protein and receptor-interacting protein kinase 1 to the cytoplasmic region via DD-DD interactions. TNF receptor-associated death domain protein further activates Fas-associated protein death domain and recruits procaspase-8 to form the death-inducing signaling complex, ultimately triggering apoptotic cascades (38, 39). TRAF-binding receptor signaling network: Upon recruitment via TIM motifs, TRAF proteins activate multiple pathways, including NF-κB, c-Jun N-terminal kinase (JNK), extracellular signal-regulated kinase (ERK), p38 activated protein kinase (p38 MAPK), and phosphatidylinositol 3-kinase (PI3K), regulating cell survival, proliferation, and cytokine secretion. 

## 4 TNF superfamily

### 4.1 CD40L-CD40

CD40 (also termed TNFRSF5), a costimulatory receptor molecule, is expressed not only on APCs such as B cells, monocytes/macrophages, and DCs, but also detected in activated T cells, endothelial cells, vascular smooth muscle cells, fibroblasts, platelets, and various epithelial lineages (40). CD40 ligand (CD40L; TNFSF5; CD154) is predominantly expressed on activated CD4^+^ T lymphocytes and platelets, with its soluble isoform (sCD40L) primarily derived from platelet secretion. This trimeric protein mediates critical bidirectional signaling between immune and vascular cells during inflammatory responses (41). While CD40 serves as the primary receptor for CD40L, this ligand demonstrates additional binding capabilities with integrin family members. The interaction between CD40L and CD40 acts as a master regulator in immunity, orchestrating T-cell activation, facilitating B-cell class-switch recombination, and modulating platelet-derived sCD40L-mediated thrombus stabilization. However, since CD40 itself lacks intrinsic enzymatic activity for signal transduction, its activation depends on the recruitment of TRAFs to mediate downstream signaling. This process activates pathways such as ​NF-κB, ​JNK, and p38 MAPK (42). Studies demonstrate that CD40L blockade transiently reduces early infiltration of T cells and macrophages, suppresses endothelial expression of adhesion receptors, and inhibits the production of E-selectin, P-selectin, and intercellular adhesion molecule-1 (ICAM-1) in endothelial cells (43). Mice with CD40 deficiency exhibit diminished neointima formation and attenuated luminal stenosis, accompanied by reduced monocyte/macrophage accumulation, inhibition of NF-κB activation, and downregulation of pro-inflammatory mediators such as ICAM-1, VCAM-1, MCP-1, matrix metalloproteinase-9 (MMP-9) and tissue factor (44). Furthermore, sCD40L induces endothelial dysfunction, promotes monocyte adhesion, exacerbates lipid deposition, and accelerates foam cell formation (45–47).

Platelet-expressed CD40, CD40L, and secreted sCD40L play pivotal roles in inflammatory responses and thrombus formation. The CD40-CD40L axis amplifies interactions among platelets, leukocytes, and endothelial cells, thereby driving leukocyte activation, endothelial recruitment/activation, and T-cell homeostasis disruption, which collectively exacerbate atherosclerosis (48, 49). Upon contact with injured vascular walls, platelets upregulate CD40L expression and release sCD40L, activating endothelial cells to induce VCAM-1 expression and IL-8 secretion (50). sCD40L modulates platelet-dependent inflammatory and thrombotic responses by enhancing platelet activation, aggregation, platelet-leukocyte interactions, and reactive oxygen/nitrogen species release, promoting atherogenesis and thrombosis (51, 52). In VSMCs, CD40-CD40L signaling contributes to neointima formation, stimulates VSMC proliferation/migration, and promotes monocyte activation, thereby accelerating intimal hyperplasia and atherosclerosis progression (44, 53, 54). Additionally, CD40L-expressing microparticles, found within atherosclerotic plaques, interact with CD40, thereby stimulating endothelial cell proliferation and *in vivo* angiogenesis (55). CD40L also promotes monocyte arrest and transendothelial migration (56). The effects of CD40-CD40L on other cell types will be elaborated in subsequent sections.

In atherosclerotic lesions, CD40 expression is significantly upregulated on intimal endothelial cells, foam cells, macrophages, dendritic cells, and smooth muscle cells, especially in carotid plaque core regions and lesion-prone areas (e.g., vascular bifurcations). Elevated CD40 mRNA and protein levels have been observed in these regions (47, 57–60). sCD40L levels strongly correlate with ASCVD and show marked elevations in patients with atherosclerosis, myocardial infarction (MI), and acute coronary syndrome (ACS) (61–64). Elevated sCD40L may indicate plaque instability or rupture, angiographic severity progression, and predicts increased cardiovascular event risk in asymptomatic carotid plaque patients, acute coronary events, and coronary restenosis (65–69). It serves as a stratification marker for systemic atherosclerosis (70). Higher sCD40L levels are associated with increased vascular lesion length and number (64, 71). Regional variations exist, with coronary ostium blood showing significantly higher sCD40L concentrations than peripheral blood, and carotid/coronary regions exhibiting elevated levels compared to renal/lower extremity territories (72, 73). sCD40L levels correlate with thrombogenesis, serving as a specific platelet activation marker that may trigger large-artery atherosclerosis-related ischemic stroke (74). Strong correlations exist between sCD40L and peak thrombin generation/thrombin generation area under the curve, with elevated circulating sCD40L independently predicting high thrombus burden in infarct-related arteries (75, 76). Genetic analyses reveal that CD40 polymorphisms (rs1535045, rs4810485, rs4239702[C]-rs1535045[T], and rs1883832 C allele) are associated with increased overall atherosclerosis risk (77–79). The CD40-1C allele (-1C/T polymorphism) correlates with unstable coronary atherosclerotic plaques, where increased C allele frequency elevates plaque rupture risk (80, 81). Anti-AS therapeutics demonstrate CD40/CD40L inhibitory effects. Atorvastatin treatment in coronary artery disease (CAD) patients significantly reduces monocyte/neutrophil surface CD40L expression and lowers sCD40L levels (82, 83). Clopidogrel-aspirin dual antiplatelet therapy in stable CAD patients attenuates platelet sCD40L release without affecting post-vascular injury thrombin generation, while clopidogrel monotherapy similarly reduces sCD40L (84–86). The antihypertensive nifedipine downregulates CD40L/sCD40L signaling in activated platelets (87).

Multiple studies demonstrate that anti-CD40L antibodies or CD154 knockout (KO) effectively suppress inflammatory responses and inhibit atherosclerosis progression in *ApoE* ^-/-^ mice. Notably, in *CD154* ^-/-^ *ApoE* ^-/-^ mice, while early lesion development remains unaffected, advanced plaques exhibit a phenotype characterized by reduced lipid content, increased collagen deposition, enhanced stability, and diminished T-lymphocyte and macrophage infiltration (88, 89). Similar conclusions were observed in *Ldlr* ^-/-^ mice treated with anti-CD40L antibodies (90). Importantly, even after advanced plaque formation, administration of anti-CD40L antibodies can induce the transformation of lipid-rich atherosclerotic plaques into stable, lipid-poor, collagen-rich plaques (91). Furthermore, siRNA-mediated CD40 silencing or CD40 KO also significantly reduces atherosclerotic lesion expansion and severity (47, 92).

The CD40-CD40L axis regulates multiple cell types, thereby influencing the pathogenesis of atherosclerosis. Endothelial cell-specific CD40 deletion significantly ameliorates plaque lipid deposition and macrophage accumulation in *ApoE* ^−/−^ mice, reduces the expression of VCAM-1 and ICAM-1, and consequently attenuates leukocyte-endothelial adhesion (93). However, this deletion increases intimal smooth muscle cell (SMC) and collagen content without altering the overall size of atherosclerotic lesions. In mice receiving *CD40* ^-/-^ *ApoE* ^-/-^ platelets, plaque progression is slower (>2-fold reduction in severity), with decreased macrophage and neutrophil infiltration, smaller lipid cores, and reduced collagen content (48). Macrophage-specific CD40 deficiency (*CD40* ^*mac *-/-^) limits atherosclerosis and systemic inflammation by suppressing pro-inflammatory macrophage polarization (94). Analysis of lymph nodes reveals reduced mRNA levels of the inflammatory chemokines C-C motif chemokine ligand 5 (CCL5) and C-C motif chemokine receptor 5 (CCR5), TNF and IL-17A, as well as the anti-inflammatory marker IL-10 in *CD40* ^*mac *−/−^ mice. This is accompanied by lowered plasma TNF-α levels, smaller plaque areas, reduced necrotic cores, and decreased lipid content. Follicular B cell-specific CD40 deletion in *Ldlr* ^-/-^ mice reduces atherosclerosis, IgG production, splenic germinal center B cells, and plasma cells. Atherogenic IgG promotes plaque progression by enhancing apoptosis/necrosis and inflammatory signaling (95). However, studies by Smook and Bavendiek et al. (96, 97)demonstrate that bone marrow-derived CD40L deficiency in *Ldlr* ^-/-^ mice alters CD25^+^ CD4^+^ T cell populations without affecting aortic arch atherosclerosis, implicating potential roles of non-hematopoietic CD40L. This conclusion is limited by artifacts from bone marrow transplantation, including sublethal irradiation-induced stromal damage and mature immune dysfunction (98). Furthermore, Lacy et al. (98) show that T cell-specific CD40L deficiency (*Cd40l* ^fl/fl^/*Cd4Cre* ^tg^) in *ApoE* ^-/-^ mice attenuates atherosclerosis, stabilizes plaques, reduces oxLDL-specific IgG (particularly IgG2b), and suppresses Th1 responses. The Th1/IFN-γ pathway is linked to T cell-dendritic cell CD40L-CD40 interactions. The absence of CD40L on platelets significantly modulates atherothrombotic processes. Repeated infusions of thrombin-activated CD40L-deficient platelets reduce leukocyte recruitment, suppress platelet-leukocyte aggregate formation, and inhibit thrombosis, thereby attenuating atherosclerosis progression (49).

Selective blockade of CD40-TRAF6 interaction preserves CD40-mediated immune functions by maintaining intact CD40-TRAF2/3/5 interactions, thereby overcoming the limitations of long-term CD40 inhibition in atherosclerosis therapy. Such an approach is achieved by TRAF-STOPs (TRAF6-specific inhibitors). Seijkens et al. (99) demonstrated that TRAF-STOP treatment in young *ApoE* ^-/-^ mice reduces classical monocyte recruitment by downregulating CD40 and β2-integrin expression, thereby attenuating atherosclerosis development. Specifically, this treatment halts plaque progression while enhancing collagen content, reducing necrotic core size, and diminishing immune cell infiltration. Furthermore, in another study, *ApoE* ^-/-^ mice with defective CD40-TRAF6 signaling exhibit attenuated atherosclerosis. This is characterized by decreased Ly6C^high^ monocyte counts, impaired recruitment of Ly6C^+^ monocytes to arterial walls, and the presence of M2-polarized macrophages with anti-inflammatory properties (100). Preclinical studies on co-stimulatory molecules of the TNF superfamily in atherosclerosis are presented in (**Table 1**).

**Table 1:** Preclinical studies on co-stimulatory molecules of the TNF superfamily in atherosclerosis.

Reference	Ligand	Receptor	Experimental model	Effect of lesions	Changes in cells	Other related changes	Effect on Atherosclerosis
(88)	CD40L	CD40	*Ldlr* ^−/−^ (HCD for 12 weeks)	AA: plaque area, wall thickness ↓ abdominal aorta: lipid deposition ↓	AA lesions: T cells, macrophages ↓	AA lesions: VCAM-1 ↓	Atheroprotective (antagonist-CD40L)
(89)			*CD40L* ^−/−^ *ApoE* ^−/−^ (diet not stated)	AA: plaque area, number of initial lesions, number of advanced lesions, calcified area, lipid core ↓, fibrous cap thickness, collagen content, αSMA ↑	lesions: VSMCs ↑, T cells, macrophages ↓	–	Atheroprotective (CD40L-deficiency)
(91)			*ApoE* ^−/−^ (chow diet)	AA: lipid core area ↓, fibrous cap thickness, collagen content, αSMA ↑	AA lesions: T cells, macrophages ↓, VSMCs ↑	AA lesions: TGF-β1 ↑	Atheroprotective (antagonist-CD40L)
(96)			*CD40L* ^−/−^ *Ldlr* ^−/−^ (HFD for 20 weeks)	no effect	peripheral blood, spleen and lymph nodes: Tregs ↓	–	No effect (Bone marrow reconstruction with CD40L-deficiency)
(97)			*CD40L* ^−/−^ *Ldlr* ^−/−^ (HCD for 16 weeks)	abdominal aorta: lipid deposition ↓	–	–	Atheroprotective (Bone marrow reconstruction with CD40L-deficiency)
			*CD40L* ^−/−^ *Ldlr* ^−/−^ (HCD for 16 weeks)	AR: plaque area ↓ AA: plaque area ↓, collagen content, αSMA ↑ abdominal aorta: lipid deposition ↓	AA lesions: macrophages ↓, SMCs ↑	–	Atheroprotective (CD40L-deficiency)
(100)			*CD40* ^−/−^ *ApoE* ^−/−^ (chow diet)	AA and thoraco-abdominal aorta: plaque area, lipid core area ↓, collagen I and II, αSMA ↑	AA lesions: macrophages, percentage of CD3^+^ T cells and CD45^+^ cells ↓, SMCs ↑ spleen: effector memory CD4^+^ and CD8^+^ T cells CD11c^+^CD4^−^CD8^−^ and plasmacytoid DCs ↓, naive CD4^+^ and CD8^+^ T cells ↑	AA lesions: MMP-2, MMP-9 ↓	Atheroprotective (CD40-deficiency)
			bone marrow-derived *CD40* ^−/−^ macrophages (stimulated by CD40-clustering antibody FGK45)	–	M1 polarization ↓ M2 polarization ↑	TIMP-1, IL-10 ↑ gelatinase activity, migration, IκBα, iNOS, IL-12 ↓	Atheroprotective (CD40-deficiency)
			*CD40L* ^−/−^ *Ldlr* ^−/−^ (HFD for 24 weeks)	AA and thoraco-abdominal aorta: plaque area ↓, collagen content, αSMA ↑	AA lesions: macrophages, percentage of CD3^+^ T cells and CD45^+^ cells ↓, SMCs ↑	–	Atheroprotective (CD40-deficiency)
			*CD40-TRAF6* ^−/−^ (chow diet)	AA and thoraco-abdominal aorta: plaque area ↓ collagen I and II, αSMA ↓	AA lesions: macrophages, percentage of CD3^+^ T cells and CD45^+^ cells ↓ peripheral blood: Ly6C^high^ monocytes, adhesion of circulating leukocytes to carotid arteries ↓ spleen: CD4^+^CD44^high^CD62L^low^ promigratory effector memory T lymphocytes, plasmacytoid DCs ↓	AA lesions: MMP-2, MMP-9 ↓	Atheroprotective (TRAF6-deficiency)
			bone marrow-derived *TRAF6* ^−/−^ macrophages (stimulated by CD40-clustering antibody FGK45)	–	M1 polarization ↓ M2 polarization ↑	TIMP-1, IL-10 ↑ gelatinase activity, migration, IκBα, iNOS, IL-12 ↓	Atheroprotective (TRAF6-deficiency)
(92)			*ApoE* ^−/−^ with CD40 gene silencing (WD diet for 16 weeks)	AS, en face aorta and ascending aorta: plaque area ↓	aorta lesions: macrophages, galectin-3^+^ macrophages ↓ intima: NF-κB^+^ cells ↓ spleen: CD40^+^ T cells, CD40^+^ monocytes ↓ ascending aorta: Taf3^+^ cells, Xpr1^+^ cells ↓	aorta: Xpr1, Taf3, miR-125b-5p, miR-30a expression ↓	Atheroprotective (silence CD40 with a specific siRNA)
(48)			*ApoE* ^−/−^ (WD diet for 4 weeks)	AA and ascending aorta: plaque area ↓ lesions: lipid core area, collagen content ↓	plasma: Ly6G^+^ neutrophils ↓ lesions: macrophages, neutrophils ↓ platelets and leukocytes adhesion to the endothelium ↓	VCAM-1 ↓	Atheroprotective (injected with *CD40* ^−/−^ *ApoE* ^−/−^ platelets)
			splenocytes from *ApoE* ^−/−^ mice	–	formation of CD45^+^CD41^+^ platelet-leukocyte aggregation ↓ platelet interactions with monocytes, DCs, and neutrophils ↓	IL-1β ↓	(coculture with *CD40* ^−/−^ *ApoE* ^−/−^ platelets)
			ECs (stimulated by TNF-α)	–	–	VCAM-1, VE-cadherin, P-selectin, platelet endothelial cell intercellular adhesion molecule-1 mRNA ↓	(coculture with *CD40* ^−/−^ *ApoE* ^−/−^ platelets)
			ex vivo model of collagen-induced thrombus formation	–	adherent leukocytes in the thrombus ↓	–	(add *CD40* ^−/−^ *ApoE* ^−/−^ or *CD40L* ^−/−^ *ApoE* ^−/−^ platelets)
(95)			*Ldlr* ^−/−^ (HFD for 8 weeks)	AS: plaque area ↓	germinal center: CD19^+^IgD^−^GL7^+^B cells ↓ spleen: plasma cells, CD44^+^PD-1^+^Bcl6^+^CD4 Tfh cells, CD44^hi^CD4^+^ T cells, CD4^+^IFN-γ^+^ T cells ↓	plasma: Ig ↓, IgG ↓	Atheroprotective (generation of B cell-specific CD40-deficient mice via bone marrow reconstruction)
(99)			*ApoE* ^−/−^ (chow diet)	AA: plaque area, necrotic core area ↓, αSMC, collagen content, initiation delay ↑	Lesions: CD3^+^ T cells, macrophages, neutrophils, macrophage proliferation ↓	–	Atheroprotective (block CD40-TRAF6 interaction)
			*ApoE* ^−/−^ (HFD for 6 weeks)	–	recruitment of leukocytes to the carotid arterial wall ↓	–	Atheroprotective (block CD40-TRAF6 interaction)
			bone marrow-derived macrophages (stimulated by CD40 agonist)	–	foam cells formation ↓	CD36, oxLDL uptake, TNF-α, IL-1β, IL-6, IL-10, IL-12, iNOS, CCL2-CCR2, CCL5-CCR5, pTak1, pNF-κB p65 ↓	Atheroprotective (block CD40-TRAF6 interaction)
(93)			*ApoE* ^−/−^ with EC-specific CD40 deletion (chow diet)	AR: lipid deposition ↓ αSMC, collagen content ↑	AR lesions: SMCs ↑, macrophages ↓	AA lesions: MMP-13 ↓ endothelial lining of AR: VCAM-1, ICAM-1 ↓	Atheroprotective (EC-specific CD40 deletion)
			mesenteric venules of *ApoE* ^−/−^ mice with EC-specific CD40 deletion (stimulated by TNF-α)	–	leukocyte rolling, leukocyte adhesion ↓	–	Atheroprotective (EC-specific CD40 deletion)
			HUVECs and monocytes (stimulated by TNF-α and a CD40L antagonist)	–	adhesion of human monocytes to HUVECs ↓	–	Atheroprotective (antagonist-CD40L)
(47)			*CD40* ^−/−^ *ApoE* ^−/−^ (chow diet)	aorta: plaque area ↓	–	–	Atheroprotective (CD40-deficiency)
			murine carotid artery bifurcation	–	carotid artery bifurcation from *ApoE* ^−/−^ mice: adherent monocytes ↑	ULVWF multimers ↑	Pro-atherogenic (agonist-CD40)
(94)			*CD40mac* ^−/−^ (HCD for 14 weeks)	AA: plaque area, necrotic core ↓ AR: lipid content ↓	blood: monocytes, T cells, CD4^+^ effector T cells ↓, CD8^+^ naive T cells ↑ intial lesions: macrophages ↓ advanced lesions: CD206^+^cells ↑ aorta: CD206^+^CD209b^−^ macrophages ratio ↑, CD26^+^ cDC2 ratio ↓	lymph nodes: TNF-α, IL-17, IL-10, CCL5, CCR5 mRNA ↓ plasma: TNF-α ↓ transcriptome of atherosclerotic aortas: genes linked to immune pathways and inflammatory responses ↓	Atheroprotective (macrophage-specific CD40 deletion)
(135)	OX40L	OX40	*Ldlr* ^−/−^ (WD for 2, 4 or 8 weeks)	carotid artery and AR: plaque area ↓	blood: percentage of CD8^+^OX40^+^ cells, CD4^+^OX40^+^ cells ↓	serum: anti-oxLDL IgG1 ↓, anti-oxLDL IgG2a/IgG1 ratio, anti-oxLDL IgM, IL-5 ↑ isolated spleen cells: IL-4 ↓, IL-5 ↑ isolated peritoneal cells: IL-4, IL-5 ↓	Atheroprotective (antagonist-OX40L)
(139)			*OX40L* ^−/−^ *ApoE* ^−/−^ (HFD for 8 weeks)	en face aorta and AR: plaque area ↓	–	blood vessels in the adventitia, subcutaneously injected matrigel: VEGF-induced angiogenesis ↓	Atheroprotective (OX40L-deficiency)
			*ApoE* ^−/−^ (HFD for16 weeks)	AR: plaque area ↓	–	–	Atheroprotective (antagonist-OX40L)
(137)			spleen lymphocytes from *ApoE* ^−/−^ mice (HFD for 4 weeks)	–	–	NFATc1 ↓	Atheroprotective (antagonist-OX40L)
			spleen lymphocytes from *ApoE* ^−/−^ mice (HFD for 4 weeks)	–	–	NFATc1 ↑	Pro-atherogenic (agonist-OX40)
(138)			CD4^+^ T lymphocytes from the spleen of *ApoE* ^−/−^ mice	–	–	NFATc1 mRNA and NFATc1 ↑ treated with NMATc1 inhibitor: IL-4 ↓	Pro-atherogenic (agonist-OX40)
			CD4^+^ T lymphocytes from the spleen of *ApoE* ^−/−^ mice		–	NFATc1 mRNA and NFATc1 ↓	Atheroprotective (antagonist-OX40L)
(132)			CD4^+^ T lymphocytes from the spleen of C57BL/6J mice	–	–	CD4^+^OX40^+^ lymphocytes: Cyclophilin A, ROS ↑	Pro-atherogenic (agonist-OX40)
(150)	CD137L	CD137	*ApoE* ^−/−^ (chow diet)	AR: plaque area ↑	AR lesions: CD3^+^ T cells, CD8⁺ T cells ↑	aorta lesions: I-A^b^ ↑, IFN-γ, TNF-α, IL-1β, ICAM-1, mRNA ↑	Pro-atherogenic (agonist-CD137)
(165)			*CD137* ^−/−^ *ApoE* ^−/−^ (chow diet)	AA, AS and descending aorta: plaque area ↓	–	lesions: IFN-γ positive cells, TNF-α, MCP-1 ↓ aorta: IFN-γ, TNF-α, MCP-1 mRNA ↓	Atheroprotective (CD137-deficiency)
			*CD137* ^−/−^ *Ldlr* ^−/−^ (HFD for 8 weeks)	AA, AS and descending aorta: plaque area ↓	–	–	Atheroprotective (CD137-deficiency)
			ECs from *CD137* ^−/−^ mice (stimulated by TNF-α and treated by an agonistic anti-CD137 antibody)	–	–	MCP-1, IL-6, VCAM-1, ICAM-1, macrophage migration, monocyte adhesion ↓	Atheroprotective (CD137-deficiency)
			RAW264.7 macrophages or lipopolysaccharide-activated peritoneal macrophages	–	–	MCP-1, TNF-α ↑	Pro-atherogenic (agonist-CD137)
			*CD137* ^−/−^ *Ldlr* ^−/−^ (HFD for 8 weeks)	AS: plaque area ↓	–	–	Atheroprotective (Bone marrow reconstruction with CD137-deficiency)
(171)			VSMCs from the thoracic aorta of C57BL/6J mice (stimulated by TNF-α)	–	–	IL-2, IL-6, TRAF6, p-p65, NFATc1 ↑	Pro-atherogenic (agonist-CD137)
(170)			*ApoE* ^−/−^ (HFD for 10 weeks)	–	–	aorta lesions: CD31 ↑	Pro-angiogenesis (agonist-CD137)
			*ApoE* ^−/−^ (HFD for 10 weeks)	–	–	aorta lesions: CD31 ↓	Anti-angiogenesis (antagonist-CD137)
			mouse brain microvascular ECs (stimulated by TNF-α)	–	–	NFATc1, EC migration, EC tube length, EC branch number ↑	Pro-angiogenesis (agonist-CD137)
			mouse brain microvascular ECs (stimulated by TNF-α)	–	–	NFATc1, EC migration, EC tube length, EC branch number ↓	Anti-angiogenesis (antagonist-CD137)
(172)			*ApoE* ^−/−^ (HFD for 8 weeks)	thoracic aorta: calcified area ↑	–	thoracic aorta lesions: autophagosomes, LC3B, Beclin1 ↑	Pro-calcification (agonist-CD137)
			thoracic aortic VSMCs (stimulated by IL-1β, IFN-γ and TNF-α)	–	–	calcium deposition, autophagosomes, ALP activity ↑ stimulated by autophagosome: calcium deposition, bone morphogenetic protein 2 ↑	Pro-calcification (agonist-CD137)
(161)			*ApoE* ^−/−^ (diet not stated)	No change	blood: granulocytes, monocytes, percentage of CD8⁺ T cells ↑ spleen: percentage of CD8⁺ T cells, CD8⁺/CD4⁺ T cells ratio ↑, CD4⁺ T cells ↓ carotid artery lesions: CD8⁺ T cells, I-A^b+^ cells ↑	abdominal aorta: TNF-α, IL-10, IFN-γ mRNA ↑, IL-6 mRNA ↓	Pro-atherogenic (agonist-CD137)
(149)			VSMCs from the thoracic aorta of C57BL/6J (stimulated by IL-1β, IFN-γ and TNF-α)	–	–	p-JNK, LC3II, p62, autophagy activity, intracellular autophagosomes, autolysosomes ↑ autophagic flux ↓	Autophagic Flux Impairment (agonist-CD137)
(167)			*ApoE* ^−/−^ (WD for 6 weeks)	carotid artery: neointima formation ↑	–	carotid artery lesions: NFATc1, calcineurin, vimentin ↑, αSMA ↓	Pro-atherogenic (agonist-CD137)
			VSMCs from the thoracic aorta of C57BL/6J (stimulated by IL-1β, IFN-γ, CM and TNF-α)	–	–	NFATc1 expression, nuclear translocation, and activation, calcineurin, vimentin, cell migration ↑, αSMA ↓	Pro-atherogenic (agonist-CD137)
(166)			*ApoE* ^−/−^ (HFD for 12 weeks)	aorta: plaque area ↑	aorta lesions: proportion of Th17 cells ↑	–	Pro-atherogenic (exosome derived from CD137‐modified ECs)
			ECs	–	–	IL-6, pAkt, NF-КB p65 ↑	Pro-atherogenic (agonist-CD137)
(163)			*ApoE* ^−/−^ (HFD for 16 weeks)	aorta: plaque area, cracks ↑ fiber cap thickness ↓	aorta lesions: foam cells, M2 macrophages ↑	aorta lesions: Arginase-1/iNOS, IL-10, arginase-1 ↑, IL-12p70 ↓	Pro-atherogenic (agonist-CD137)
			peritoneal macrophages	–	–	iNOS, IL-12p70 ↓, Arginase-1, IL-10, p-STAT6, PPARδ ↑ fluorescence intensity: CD206 ↑, CD86 ↓	Pro-macrophage M2 polarization (agonist-CD137)
			HUVECs and MBVECs (co-cultured with supernatants from CD137-stimulated peritoneal macrophages)	–	–	MBVECs: migration ↑ HUVECs: EC tube length, EC branch number ↑	Pro-angiogenic (agonist-CD137)
(169)			*CD137* ^−/−^ *ApoE* ^−/−^ (HFD for 11-13 weeks)	aortic ring: sprout number ↓	–	aorta lesions: CD31-positive microvessels ↓	Anti-angiogenesis (CD137-deficiency)
			ECs	–	–	VEGFR2, p-Akt, p-eNOS ↑	Pro-angiogenesis (agonist-CD137)
			HUVECs and MBVECs	–	–	HUVECs: EC tube length, EC branch number ↓ MBVECs: migration, proliferation ↓	Anti-angiogenesis (silence CD137 with a specific siRNA)
(173)			*ApoE* ^−/−^ (WD for 13 weeks)	–	–	aorta lesions: Beclin 1, P62, LC3II/I, autophagosomes ↑ autolysosomes, autophagic flux ↓	Autophagic Flux Impairment (agonist-CD137)
			VSMCs from the thoracic aorta of C57BL/6J mice (stimulated by TNF-α)	–	–	Beclin 1, P62, LC3II/I, autophagosomes ↑ autolysosomes, autophagic flux, Rab7 ↓	Pro-calcification (agonist-CD137)
(187)	GITRL	GITR	*Ldlr* ^−/−^Gitr-TG (HCD for 11 weeks)	AR: plaque area ↓	lymph nodes: Tregs ↑ thymus: CD4^+^ T cells, Tregs ↑ AR lesions: CD3^+^ T cells, Tregs ↑	thymus: IL-2, IFNγ mRNA ↑ lymph nodes: IFNγ mRNA ↑	Atheroprotective (Bone marrow reconstruction with GITRL-Tg)
(185)			*Gitr* ^−/−^ *ApoE* ^−/−^ (WD)	AR: plaque area, necrotic core, vulnerability-index ↓, fibrous cap ↑	AR lesions: macrophage ↓	–	Atheroprotective (GITR-deficiency)
			classical monocytes and non-classical monocytes of *Gitr* ^−/−^ *ApoE* ^−/−^ mice	–	leucocyte recruitment ↓	non-classical monocytes: ROS, mitochondrial activation ↓ classical monocytes and non-classical monocytes: CD11b, L-selectin ↓	Atheroprotective (GITR-deficiency)
			bone marrow-derived macrophages of *Gitr* ^−/−^ *ApoE* ^−/−^ mice	–	cell migration ↓	CCL3, CCL4, CXCL2, IL-6, IL-10, IL-17A ↓ mitochondrial activation ↓	Atheroprotective (GITR-deficiency)
			*Gitr* ^−/−^ *Ldlr* ^−/−^ (WD)	lesions: necrotic core ↓, fibrous cap ↑	lesions: macrophages ↓	–	Atheroprotective (Bone marrow reconstruction with GITRL-deficiency)
(186)			*BALB/c* (HCD for 11-12weeks)	coronary artery and vascular crescent plaques: microthrombus accumulation ↓ coronary artery: plaque area ↓	blood: Th1 cells ↓	blood: TNF-α, TNF-β, IL-1β ↓ coronary artery: p-STAT1 ↓	Atheroprotective (antagonist-GITR or silence GITR with a specific siRNA)
(202)	CD70	CD27	ApoE*3-Leiden CD70-TG (HFD for 12/16/20 weeks)	AR: plaque area ↓	peripheral blood: Ly6C^hi^ CD62L^+^ monocytes ↑ spleen: percentage of B cells ↓	serum: anti-oxLDL antibodies, cholesterol ↓	Atheroprotective (CD70-Tg)
			monocytes from CD70-TG mice	–	–	activation, apoptosis susceptibility, TNF-α production ↑	Atheroprotective (CD70-Tg)
(197)			*CD70* ^−/−^ *ApoE* ^−/−^ (WD for 7 weeks)	ascending aorta: plaques area, necrotic core area ↑	AR lesions: macrophage ↑ peripheral blood: Ly6C^+^ monocytes ↑	–	Pro-atherogenic (Bone marrow reconstruction with CD70-deficiency)
			*CD70* ^−/−^ *ApoE* ^−/−^ (chow diet for 18 weeks)	ascending aorta: plaques area ↑	spleen: Tregs ↓		Pro-atherogenic (CD70-deficiency)
			bone marrow-derived macrophages from *CD70* ^−/−^ *ApoE* ^−/−^ mice	–	–	inflammatory active, metabolically active, foam cell formation capacity, scavenging capacity, cholesterol efflux capacity ↓	Pro-atherogenic (CD70-deficiency)
(203)			*CD27* ^−/−^ *ApoE* ^−/−^ (HCD for 7 weeks)	AR: plaques area, necrotic core area ↑	AR lesions: CD4^+^ T cells, Tregs ↓, macrophages ↑ spleen, aorta, lymph nodes: Tregs ↓ spleen: Tregs proliferation ↓	aortic lesions: IL-1β, IL-6, IL12p53, Gata-3, ICAM-1, VCAM-1, CCL1 mRNA ↑ plasma: TGF-β ↓	Pro-atherogenic (Bone marrow reconstruction with CD27-deficiency)
			*CD27* ^−/−^ *ApoE* ^−/−^ (chow diet)	AR: plaques area ↑	lesions: macrophages ↑ spleen, lymph nodes, aorta, blood: Tregs ↓	–	Pro-atherogenic (CD27-deficiency)
(211)	CD30L	CD30	*Ldlr* ^−/−^ (WD for 8 weeks)	AR: plaque area ↓	spleen and mediastinal heart lymph nodes: CD4^+^ T cells ↓ spleen: CD4^+^ T cells proliferation ↓ adventitia: T cells ↓	–	Atheroprotective (antagonist -CD30L)
(232)	TL1A	DR3	*ApoE* ^−/−^ (HFD for 12 weeks)	descending aorta, thoracic aorta, and abdominal aorta: plaque area ↓ AR: plaque area, necrotic core area, calcified area ↓, fibrous cap thickness, collagen content, αSMA ↑	–	aorta lesions: osteopontin, runt-related transcription factor 2, ALP, MMP9 ↓, ABCG1 ↑	Atheroprotective (agonist-DR3)
			human primary aortic smooth muscle cells	–	human aortic smooth muscle cells/foam cells ↓	runt-related transcription factor 2, liver X receptor α, liver X receptor β, p53, smooth muscle protein 22α, αSMA, ALP, ABCA1, ABCG1, cholesterol efflux ↑ serum response factor, myocardin, miR-203-3p mRNA ↑ osteopontin, epiregulin, msh homeobox 2 mRNA ↓	Atheroprotective (agonist-DR3)
(247)	LIGHT/LTα_1_β_2_	LTβR/HVEM	*TNFR1* ^−/−^ *TNFR2* ^−/−^ macrophages	–	–	macrophage: CXCL13, ABCA1 ↑	Atheroprotective (agonist-LTβR)
(279)	BAFF	BAFFR/BCMA/TACI	*BAFF-R* ^−/−^ *Ldlr* ^−/−^ (HFD for 6 or 8 weeks)	AR: plaque area ↓	bone marrow, blood, spleen, and lymph nodes: B cells ↓ percentages of peritoneal B-cell subsets: B1b cells, B2 cells ↓ AR lesions: macrophage accumulation, T cells ↓	plasma: anti-MDA-LDL IgG1, anti-MDA-LDL IgG2c ↓	Atheroprotective (Bone marrow reconstruction with BAFFR-deficiency)
(23)			*BAFF-R* ^−/−^ *ApoE* ^−/−^ (HFD for 8 weeks)	AR: plaque area ↓	blood, peritoneal cavity, spleen and lymph nodes: CD22^+^ B cells ↓ peritoneal cavity: B2 cells ↓ AR lesions: macrophages accumulation, CD4^+^ T cells, CD8^+^ T cells, PCNA^+^ cells, CD11c^+^ DCs, CD83^+^ DCs ↓	AR lesions: VCAM-1 ↓ TNF-α, IL-1β, MCP-1 mRNA ↓ AR lesions and plasma: IgG1, IgG2a, IgM ↓	Atheroprotective (BAFFR-deficiency)
(277)			*ApoE* ^−/−^BAFF-Tg (atherogenic diet for 8 or 12 weeks)	AR: plaque area ↓	–	serum: cholesterol ↓, very low-density lipoprotein peak ↓, anti-PC IgM, anti-MDA-LDL IgM, anti-PC IgG, anti-MDA-LDL IgG ↑ (IgG2b, IgG2c significant ↑)	Atheroprotective (BAFF-Tg)
			*Taci* ^−/−^ *ApoE* ^−/−^BAFF-Tg (atherogenic diet for 8 or 12 weeks)	AR: plaque area ↑	–	serum: cholesterol ↑, anti-PC IgM, anti-MDA-LDL IgM ↓	Pro-atherogenic (BAFF-Tg and TACI-deficiency)
(271)			*ApoE* ^−/−^ (atherogenic diet for 6 or 8 weeks)	AR: plaque area, necrotic core area, cleaved caspase-3 ↑ collagen content ↓	blood: B2 cells ↓, Ly6C^high^ monocytes ↑ spleen: B1 cells ↓ peritoneal: CD23^+^ B2 cells ↓	serum: KC, MCP-1 ↑ plasma: Ig, IgM, IgG1, IgG2c, IgG3, anti-MDA-LDL IgG ↓	Pro-atherogenic (antagonist -BAFF)
			*Taci* ^−/−^ *ApoE* ^−/−^ (atherogenic diet for 6 weeks)	AR: plaque area ↑	AR lesions: macrophages ↑	–	Pro-atherogenic (TACI-deficiency)
(280)	APRIL	BCMA/TACI	*ApoE* ^−/−^APRIL-Tg (WD for 12 weeks)	AR: αSMA ↑	peritoneal: percentages of B cells, B1a cells, B1b cells, concentration of B1a cells ↑, concentration of B2 cells ↓ AR lesions: SMCs ↑	lesions: IgM ↑ plasma: IgM, IgG anti-MDA-LDL IgM, anti-CuOx-LDL IgM ↑	Pro-stabilizing (APRIL-Tg)
(273)			*APRIL* ^−/−^ *Ldlr* ^−/−^ (atherogenic diet for 10 weeks)	AR: plaque area, necrotic core area, acellular areas ↑	AR lesions: macrophage ↑	plasma: Ig ↓ plaque: ApoB ↑	Pro-atherogenic (APRIL-deficiency)

Up arrows indicate increases, and down arrows indicate decreases.

AA, aortic arch; αSMA, alpha smooth muscle actin; ApoE, apolipoprotein E; AR, aortic root; AS, aortic sinus; BMT, BM transplantation; Cc11, Eotaxin-1; CCL2, C-C Motif Chemokine Ligand 2; CCL3, C-C Motif Chemokine Ligand 3; CCL4, C-C Motif Chemokine Ligand 4; CCL5, C-C Motif Chemokine Ligand 5; CCR2, C-C Chemokine Receptor 2; CCR5, C-C Chemokine Receptor 5; CypA, Cyclophilin A; CuOx-LDL, CuSO4-oxidized low-density lipoprotein; CXCL, C-X-C Motif Chemokine Ligand; EC, endothelial cell; DC, dendritic cell; fl/fl, “floxed” gene; E-selectin, Endothelial-selectin; F4/80, EGF-like module-containing mucin-like hormone receptor; HCD, High-Cholesterol Diet; Gata-3, GATA-binding protein 3; HFD, high fat food diet,containing 21% fat and 0.15% cholesterol; HDL, high-density lipoprotein; HVEM, herpes virus entry mediator; ICAM-1, intercellular adhesion molecule-1^*^; iEC, intestinal epithelial cell; IgG, Immunoglobulin G; IgM, Immunoglobulin M; Ikbα, inhibitor of nuclear factor kappa B alpha; Ikkβ, Inhibitor of Nuclear Factor Kappa B kinase beta subunit; IL-1β, Interleukin-1 Beta; IL-4, Interleukin-4; IL-5, Interleukin-5; IL-6, Interleukin-6; IL-8, Interleukin-8; IL-10, Interleukin-10; IL-12, Interleukin-12; IL-12p70, Interleukin-12 p70; IL-17A, Interleukin-17A; IL-18, Interleukin-18; iNOS, Inducible nitric oxide synthase; I-A^b+^ cells, MHC class II I-A^b+^ positive cells; KC, keratinocyte chemoattractant; Ldlr, low-density lipoprotein receptor; LC3B, Microtubule-associated protein 1A/1B-light chain 3B; M1 macrophages, Classically activated macrophages; M2 macrophages, alternatively activated macrophages; Mac-2^+^, Galectin-3; Mac-3^+^, Macrosialin; MCP-1, monocyte chemoattractant protein-1; MDA-LDL, malondialdehyde conjugated with low-density lipoprotein; MMP-2, Matrix Metalloproteinase-2^†^; MMP-9, Matrix Metalloproteinase-9^†^; MMP-13, Matrix Metalloproteinase-13^†^; MMP-14, Matrix Metalloproteinase-14^†^; MHCII, Major Histocompatibility Complex class II molecule; NF-κB, nuclear factor kappa-light-chain-enhancer of activated B cells; NMATc1, Nuclear factor of activated T-cells 1; NKs, Natural Killer cells; NKTs, Natural Killer cells; PCNA, Proliferating cell nuclear antigen; pDC, plasmacytoid dendritic cell; P-selectin, Platelet-selectin; ROS, Reactive Oxygen Species; SMC, smooth muscle cell; Tg, transgenic—knock-in to induce overexpression; TGF-β1, transforming growth factor beta1; Th1 cells, T helper 17 cells; Th17 cells, T helper 17 cells; TNF, tumor necrosis factor; TNF-α, tumor necrosis factor-alpha; Tregs, regulatory T cells; TRAF, tumor necrosis factor receptor-associated factor; ULVWF, Ultra-large von Willebrand factor; VCAM-1, vascular cell adhesion molecule-1; VEGF, Vascular Endothelial Growth Factor; VSMCs,Vessel smooth-muscle-cells; WD, Western diet,a high-fat, high-cholesterol diet formation to accelerate atherosclerosis; *https://www.ncbi.nlm.nih.gov/mesh/68018799 †https://www.ncbi.nlm.nih.gov/mesh/68020780

**Figure 3:**
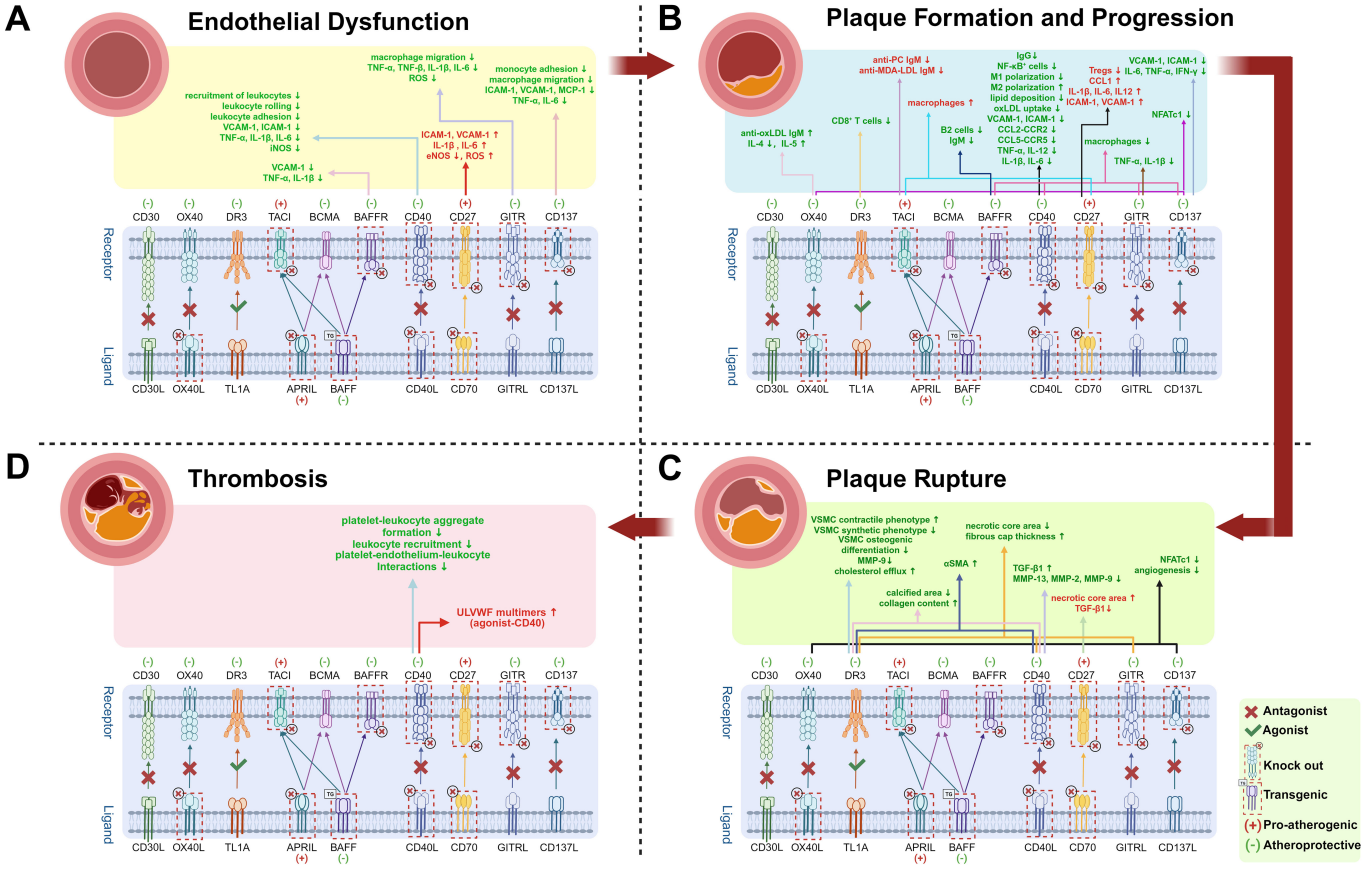
Effects of TNF superfamily member knockout or antagonists in Atherosclerosis. Up arrows (↑) indicate increases, and down arrows (↓) indicate decreases. Red “(+)” and text denote pro-atherogenic effects; green “(−)” and text denote atheroprotective effects. Text in red highlights molecules or cells that promote atherosclerosis; green text indicates inhibition. Atherosclerosis progresses through four stages. **(A)** Endothelial Dysfunction: Partial TNF superfamily knockout or antagonists reduce chemokine expression, including vascular cell adhesion molecule-1 (VCAM-1), intercellular adhesion molecule-1 (ICAM-1), and monocyte chemoattractant protein-1 (MCP-1), thereby suppressing leukocyte recruitment, rolling, and adhesion. Downregulated tumor necrosis factor-alpha (TNF-α), interleukin-1 beta (IL-1β), interleukin-6 (IL-6), and reactive oxygen species (ROS) production mitigate endothelial inflammation and oxidative stress; **(B)** Plaque Formation and Progression: Inhibiting pathways such as CD40, GITR, and CD137 decreases macrophage infiltration and downregulates chemokine and pro-inflammatory cytokines expression. Elevated anti-oxidized low-density lipoprotein immunoglobulin M (anti-oxLDL IgM) levels confer atheroprotection. CD40-CD40L blockade further drives M1-to-M2 macrophage polarization (M1/M2 polarization), shifting pro-inflammatory macrophages toward an anti-inflammatory phenotype. Nuclear factor of activated T cells; cytoplasmic 1 (NFATC1) promotes inflammation by regulating pro-inflammatory cytokine production; **(C)** Plaque Rupture: Inhibition of CD40 and GITR pathways stabilizes plaques-characterized by increased collagen content, thicker fibrous caps, smaller necrotic cores, reduced calcification, and higher numbers of αSMA-positive smooth muscle cells (SMCs). Pathway inhibition also reduces secretion of matrix metalloproteinase-9 (MMP-9), MMP-2, and MMP-13, thereby preserving fibrous cap integrity. Additionally, blocking CD137 and OX40-related pathways suppresses neovascularization and can further reduce NFATC1, which modulates SMC proliferation and calcification;. **(D)** Thrombosis: Activation of CD40 mediates elevated ultra-large von Willebrand factor (ULVWF) levels, increasing thrombotic risk. Conversely, inhibition of the CD40-CD40L pathway suppresses thrombosis by reducing platelet-leukocyte aggregate formation, leukocyte recruitment, and platelet-endothelium-leukocyte interactions-thereby lowering the risk of atherosclerosis complications such as stroke. Created by https://BioRender.com. (Complete data are shown in **Table 1**).

Notably, platelet-derived CD40 and CD40L play pivotal roles in plaque thrombosis and atherosclerosis progression. Although CD40/CD40L signaling inhibition in distinct cell types exerts anti-atherosclerotic effects, the underlying mechanisms vary, reflecting the complexity of immune networks in AS and necessitating further mechanistic studies. Elevated sCD40L levels not only indicate plaque presence but also correlate with plaque instability and increased cardiovascular risk, highlighting its potential as a diagnostic biomarker. Studies on TRAF-STOPs (TRAF6-specific inhibitors) demonstrate that targeting downstream signaling pathways significantly reduces immune activation, providing a rationale for developing novel antagonists. Currently, multiple CD40L-CD40 agonists and antagonists are under clinical investigation. Recent *in vivo* studies reveal that CD40 agonists exhibit remarkable efficacy in cancer immunotherapy, particularly when combined with immune checkpoint inhibitors. However, the CD40L-CD40 axis is also critically involved in cardiovascular pathologies such as AS (**Figure 3**). Thus, optimizing dosage regimens and managing adverse effects (e.g., cardiovascular toxicity) are essential in future CD40 agonist-based cancer therapies. Additionally, CD40L/CD40 antagonists show therapeutic potential for autoimmune diseases, including Sjögren’s syndrome (SjD) and systemic lupus erythematosus (SLE). In a Phase II trial involving patients with SjD, the CD40L antagonist dazodalibep significantly reduced systemic disease activity while demonstrating a favorable safety and tolerability profile. Treatment also led to a significant reduction in serum levels of the chemokine CXCL13, a biomarker linked to disease activity (101). A separate Phase II trial assessed frexalimab, a second-generation anti-CD40L antibody engineered to lack the FcγRIIA-activating domain (mitigating thrombotic risk), in patients with relapsing multiple sclerosis, demonstrating favorable efficacy (102). Separately, *post hoc* analyses of studies involving the anti-CD40 monoclonal antibody BI 655064 suggest a potential clinical benefit for patients with active lupus nephritis (103). While next-generation agents exhibit improved safety profiles, further optimization of drug design and exploration of combination therapies remain imperative. The dual role of the CD40-CD40L axis in both immune activation and cardiovascular pathologies underscores the need for carefully balanced therapeutic approaches. Future research should focus on refining these agents to maximize their therapeutic benefits while minimizing potential risks.

### 4.2 OX40L-OX40

OX40L (TNFSF4) is the ligand for OX40 (TNFRSF4), a member of the TNF superfamily. OX40 is primarily expressed on activated T cells, while OX40L is expressed in various immune cells, including macrophages, mast cells, DCs, B cells, and vascular endothelial cells (104). As a critical co-stimulatory molecule for T cell signaling, it plays a significant role in promoting T cell proliferation and differentiation, mediating adhesion between T cells and endothelial cells, and regulating the antigen-presenting function of macrophages (105). OX40L also modulates downstream signaling pathways such as NF-κB, PI3K/Akt, and AP-1, which are involved in inflammatory responses and fibrosis (106).

Specifically, at the T cell level, this pathway promotes the survival of CD4^+^ T cells at inflammatory sites, and collaborates with TNF signaling to upregulate TNFR2 expression on Treg cells, optimizing their activation and thereby enhancing Treg-mediated inflammatory suppression (107). The study revealed that agonist administration boosts Treg proliferation, while Treg cells deficient in OX40 show decreased survival within inflamed tissues (108, 109). In antigen presentation, OX40L on dendritic cells can induce inflammatory Th2 differentiation (110). Platelet OX40L expression correlates positively with serum MMP-9 and MMP-3 levels, suggesting its role in regulating plaque instability (111). Studies in human umbilical vein endothelial cells demonstrate that TNFSF4 (OX40L) knockdown effectively reverses endothelial cell apoptosis, oxidative stress, and vascular dysfunction caused by oxLDL (112). Further research reveals that oxLDL-induced upregulation of OX40L in endothelial cells is associated with increased expression of the oxidized low-density lipoprotein receptor lectin-like oxidized low-density lipoprotein receptor-1 (113). In the tumor microenvironment, OX40 activation on tumor endothelial cells promotes tumor immune evasion through S1P/YAP-mediated angiogenesis (114). Additionally, OX40^+^ T cells drive B cell isotype switching through ligand-receptor interactions; blocking this interaction significantly reduces the anti-hapten IgG response, while the IgM response remains largely unaffected (115). This signaling axis also amplifies inflammatory responses by enhancing IFN-γ, TNF-α secretion, and perforin release, upregulating IL-2 and IFN-γ expression, and inducing the production of chemokines CCL2 and CCL5, thereby forming a multi-layered inflammatory amplification network (116–119).

The expression of OX40/OX40L and plasma levels of sOX40L are closely associated with ASCVD. Studies demonstrate that sOX40L levels are significantly elevated in patients with ACS (111, 120) and are markedly higher in unstable angina (UA) and acute myocardial infarction (AMI) patients compared to stable angina patients and healthy controls (120, 121). Elevated sOX40L levels are positively correlated with increased carotid intima-media thickness, plaque instability, severity of coronary artery stenosis, and heightened risk of cardiovascular events (121–123). Additionally, patients with severe cerebral infarction exhibit significantly higher sOX40L levels than healthy controls, and non-survivors show higher serum OX40L levels than survivors (124). At the genetic level, both OX40 and OX40L mRNA and protein expression are upregulated in coronary heart disease (CHD) patients compared to healthy individuals (125). In ACS patients, elevated OX40L expression and its mRNA/protein levels are positively associated with coronary stenosis, atherosclerotic plaque instability, and sudden cardiac death (111, 126, 127). The role of TNFSF4 gene polymorphisms in ASCVD demonstrates significant heterogeneity. Early meta-analyses found no significant association between TNFSF4 polymorphisms (rs3850641 and rs17568) and CAD or stroke risk (128). However, subsequent analyses incorporating 18 studies indicate that rs17568, rs1234314, and rs3850641 variants may serve as genetic biomarkers for specific CAD subtypes (129). These contradictory findings underscore the disease subtype-specific and population-dependent nature of TNFSF4 polymorphisms in AS. Pharmacologically, statins exhibit immunomodulatory effects via the mevalonate pathway. Simvastatin suppresses OX40 and OX40L expression in human peripheral blood mononuclear cells and antagonizes interferon-γ-induced upregulation of OX40/OX40L mRNA and protein (130). Rosuvastatin demonstrates concentration-dependent inhibition of OX40L in vascular endothelial cells and lymphocytes, counteracting oxLDL-stimulated OX40L expression (131). Clinically, atherosclerotic cerebral infarction patients receiving simvastatin in combination with conventional therapy for six months exhibit significantly lower serum sOX40L levels than those treated with conventional therapy alone (130). These findings provide a pharmacological rationale for targeting the OX40/OX40L pathway in precision medicine approaches.

The OX40-OX40L signaling pathway promotes atherosclerotic plaque formation through multifaceted mechanisms. Studies demonstrate that this pathway contributes to plaque development by inducing elevated reactive oxygen species (ROS) and enhancing cyclophilin A secretion from lymphocytes (132). Genetic evidence reveals that the Tnfsf4 gene (encoding OX40L), located within the murine atherosclerosis susceptibility locus Ath1, is critically linked to plaque progression (133). Correspondingly, polymorphisms in the human ortholog TNFSF4 elevate MI risk, implicating its role in plaque destabilization (133). Tnfsf4-targeted mutant mice exhibit significantly smaller atherosclerotic lesions compared to controls, whereas Tnfsf4 overexpression markedly enlarges plaque area (134). Immunoregulatory studies indicate that blocking OX40-OX40L interactions suppresses IL-4 production, thereby inhibiting Th2 cell-driven B-cell isotype switching and ultimately increasing anti-atherosclerotic oxLDL-specific IgM (135). In *Ldlr* ^−/−^ mouse models, anti-OX40L therapy attenuates Th2-type immune responses anti-OX40L therapy attenuates Th2-type immune responses by reducing GATA-binding protein 3 and IL-4 levels, lowers IgE levels, and diminishes mast cell numbers and activity (136). The concurrent increase in IgM observed in these experiments is mediated by OX40L blockade-induced IL-33 secretion from APC, thereby promoting IL-5 production by T cells and B1 cells (136). Further research elucidates the pathway’s role in plaque progression via nuclear factor of activated T cells, cytoplasmic 1 (NFATc1) regulation (137). OX40-OX40L activation significantly upregulates NFATc1 mRNA and protein levels in lymphocytes of *ApoE* ^−/−^ mice, while NFATc1 inhibition effectively suppresses smooth muscle cell proliferation and attenuates plaque formation (138). Additionally, *OX40L* ^−/−^ *ApoE* ^−/−^ mice exhibit reduced aortic adventitial vascular density and impaired vascular endothelial growth factor-induced angiogenesis (139). Notably, when *ApoE* ^−/−^ mice fed a high-fat diet receive bone marrow transplants from wild-type or *OX40L* ^−/−^ donors, aortic plaque severity remains comparable between groups. This indicates that vascular OX40L (rather than bone marrow-derived OX40L) plays a dominant role in atherogenesis (139). These findings systematically delineate the molecular network through which OX40-OX40L signaling synergistically drives atherosclerosis via oxidative stress, immune response modulation, and vascular remodeling.

More than ten humanized OX40/OX40L antibodies have entered clinical trials, including both agonists and antagonists. OX40 agonists are undergoing evaluation in solid tumors and hematologic malignancies, with several studies exploring combination therapies involving OX40 and other immune checkpoint inhibitors (e.g., PD-1, CTLA-4, 4-1BB). Previous clinical studies indicate that PD-1 and CTLA-4 inhibitors exhibit cardiotoxic effects that may accelerate atherosclerosis progression. Similarly, OX40 agonism could promote atherogenesis through pro-inflammatory and pro-angiogenic mechanisms (140). Therefore, the potential impact of OX40-targeted therapies on ASCVD warrants careful monitoring in clinical trials. OX40/OX40L antagonists (e.g., Oxelumab, Amlitelimab, Cudarolimab, Vonlerolizumab, Rocatinlimab) have demonstrated efficacy in atopic dermatitis, asthma, and ulcerative colitis, with notable therapeutic success in atopic dermatitis (141). Rocatinlimab, the first anti-OX40 monoclonal antibody to successfully complete a Phase III trial, has demonstrated consistent therapeutic efficacy across multiple clinical studies in patients with moderate-to-severe atopic dermatitis. These findings position it as the potential first-in-class OX40 inhibitor to reach the global market. In its Phase IIb trial, rocatinlimab significantly improved atopic dermatitis symptoms, with most patients maintaining symptom control after treatment discontinuation. The therapy exhibited favorable tolerability and effectively inhibited Th2 T-cell activation and clonal expansion (142). Similarly, amlitelimab (a novel OX40 ligand-blocking agent) is advancing through Phase III development. Its Phase IIb trial demonstrated significant reductions in both clinical severity scores and key inflammatory biomarkers (Th2/Th17/Th22-related cytokines) among atopic dermatitis patients, alongside a safety profile. Notably, sustained clinical responses and suppression of inflammatory biomarkers persisted for weeks despite serum amlitelimab concentrations falling to low or negligible levels (143). Given the demonstrated role of OX40 inhibition in suppressing AS plaque formation, future investigations should evaluate OX40 antagonists as potential anti-atherosclerotic agents. Mentioned in the previous study, blocking OX40/OX40L interactions reduces atherosclerosis by suppressing anti-ox-LDL IgM elevation. Furthermore, rosuvastatin and simvastatin downregulate OX40 expression, indicating a synergistic relationship between lipid-lowering therapies and OX40 modulation. The combination of these agents may provide enhanced therapeutic benefits in AS management.

### 4.3 CD137L-CD137

CD137 (4-1BB), a member of the TNFRSF, is expressed on most immune cells, including activated T cells, NK cells, monocytes, neutrophils, and DCs, as well as on non-immune cells such as endothelial cells (144). Its ligand, CD137L (4-1BBL), a type II transmembrane protein, is predominantly expressed on APCs, including monocytes, macrophages, DCs, and activated B cells.

The CD137-CD137L signaling system bidirectionally regulates immune responses and atherosclerosis progression. CD137 promotes T-cell clonal expansion, differentiation, and survival, while CD137L triggers reverse signaling in APCs, inducing their activation, maturation, and enhancing their cytokine secretion and antigen-presenting capacity. This synergistically activates B cells and monocytes (145–147). By binding to TRAF1 and TRAF2, CD137 activates signaling pathways such as NF-κB, ERK, and JNK/p38 MAPK, driving pro-inflammatory cytokine production (148). It may also regulate VSMC autophagy via the JNK pathway (149). In atherosclerotic lesions, pro-inflammatory cytokines reverse induce CD137 expression on ECs and VSMCs. CD137 activation by its ligand further upregulates EC surface adhesion molecules VCAM-1 and ICAM-1, enhancing monocyte and DC recruitment to lesion sites. Circulating CD137L^+^ cells bind and activate CD137 on ECs, forming a self-reinforcing positive feedback loop that perpetuates inflammatory responses (150–152). For example, CD137 stimulation drives effector CD8^+^ T cells to accumulate in low-shear or hemodynamically disturbed regions. These T cells secrete chemokines to recruit additional CD8^+^ T cells into plaques, exacerbating intraplaque immune-inflammatory microenvironments and tissue damage (153).

Clinical studies have shown a close link between the CD137L/CD137 axis and atherosclerosis progression. Atherosclerotic lesions in patients with AS exhibit significantly elevated expression levels of CD137 and its ligand compared to normal vascular tissues (150). This is also seen in ACS patients, where both peripheral Tregs and conventional T cells show marked CD137 upregulation (154). And the intensity of CD137 expression shows a significant positive correlation with the severity of coronary stenosis and plaque instability (155). Notably, sCD137 and its membrane-bound form are elevated in patients with ACS and acute ischemic atherosclerotic stroke (156–158). Higher sCD137 levels are tied to greater cardiovascular event risks (156–158). Moreover, CD137 genetic polymorphisms are strongly linked to carotid intima-media thickening and ischemic stroke susceptibility, highlighting its potential as a key biomarker for atherosclerotic diseases (159, 160).

Administration of CD137 agonists to *ApoE* ^−/−^ mice exacerbates aortic inflammation, significantly increases atherosclerotic lesion size, and amplifies CD8^+^ T-cell infiltration within the aorta. This treatment also upregulates pro-inflammatory cytokines (e.g., IFN-γ, TNF-α, IL-1β) and adhesion molecules (e.g., ICAM-1), which are critical for leukocyte transmigration from circulation into the vascular wall and subsequent plaque formation (150, 161). An additional study confirmed the role of CD137-activated CD8^+^ T cells in AS pathogenesis. CD137 agonists mediate the activation of effector CD8^+^ T cells, driving their infiltration into low-shear stress and turbulent flow regions of the vascular intima and sustaining an innate-like pro-inflammatory program. The persistent retention of these CD137-activated CD8^+^ T cells further recruits endogenous CD8^+^ T cells with IFN-γ-producing potential, thereby amplifying intraplaque inflammation (153). In early cerebral ischemia models, CD137 co-stimulation enhances T-cell activation, exacerbating inflammatory immune responses and aggravating ischemic brain injury. Conversely, blocking the CD137/CD137L pathway mitigates post-ischemic cerebral damage, thereby highlighting its therapeutic potential (162).

In the regulation of monocytes/macrophages, CD137 signaling promotes M2 macrophage polarization via the STAT6/PPARδ pathway, a phenotypic shift that exacerbates plaque instability through pro-angiogenic effects (163, 164). Additionally, sCD137 stimulates CD137L signaling in monocytes/macrophages, enhancing the production of TNF-α and MCP-1 (165). In endothelial cells, CD137 deficiency results in impaired monocyte recruitment and adhesion, and reduces atherosclerotic plaque burden (165). In *CD137* ^-/-^ *ApoE* ^-/-^ and *CD137* ^-/-^ *Ldlr* ^-/-^ mouse models, activation of CD137 signaling using the 3H3 antibody (which mimics CD137L) promotes endothelial secretion of pro-inflammatory mediators, including VCAM-1, ICAM-1, MCP-1, and IL-6 (165). CD137/CD137L activation also significantly modulates endothelial-mediated inflammation and leukocyte adhesion. Administration of CD137-modified endothelial cell-derived exosomes in *ApoE* ^-/-^ mice significantly increases plaque area (166). This effect is linked to NF-κB signaling-driven IL-6 upregulation and subsequent IL-6-induced Th17 cell differentiation, which accelerates atherogenesis (166).

The CD137-CD137L signaling pathway exhibits multifaceted regulatory capabilities in vascular remodeling, intimal lesion development CD137-CD137L interactions activate NFATc1 in VSMCs of *ApoE* ^-/-^ mice, promoting VSMC phenotypic switching and migration, and thus contributing to neointimal lesion formation (167). Conversely, blocking the CD137/CD137L pathway effectively suppresses post-injury intimal hyperplasia (168). In terms of angiogenesis, CD137 activation promotes sprouting angiogenesis in *ApoE* ^−/−^ mice by increasing vascular endothelial growth factor A secretion and activating the VEGFR2/Akt/eNOS pathway (169). Moreover, CD137 may promote intraplaque angiogenesis via NFATc1 activation, thereby further destabilizing atherosclerotic lesions (170). Furthermore, Yan et al. (171) demonstrated that CD137 signaling regulates NFATc1 and its downstream cytokines (e.g., IL-2 and IL-6) in VSMCs through the TRAF6/NF-κB p65 pathway. In the context of calcification, CD137-CD137L activation accelerates VSMC and aortic plaque calcification in *ApoE* ^−/−^ mice (172, 173). This effect is likely mediated by p38 MAPK signaling activation, impaired autophagosome exocytosis, and inhibition of autophagic degradation (172–174).

Current research on CD137 primarily focuses on agonist-based immunotherapies for oncology. The most advanced CD137/CD137L agonists have progressed to phase III trials, but not solely targeting CD137-instead, concurrently targeting CD19 and combined with chemotherapy or other immunotherapeutic agents, as exemplified by clinical trial NCT03570892. Notably, no CD137/CD137L antagonists are currently under clinical investigation. Emerging evidence indicates that CD137 activation is implicated in atherosclerosis and the progression of ASCVD, including stroke. Specifically, CD137 agonism exacerbates critical atherosclerotic processes—such as leukocyte aggregation, lipid core expansion, pathological neovascularization, and calcification—thereby enhancing plaque vulnerability. Consequently, pharmacological inhibition of the CD137 pathway may represent a novel therapeutic strategy for stabilizing vulnerable plaques, particularly in advanced atherosclerotic patients with active inflammation.

### 4.4 GITRL-GITR

Glucocorticoid-induced tumor necrosis factor receptor family-related protein (GITR; TNFRSF18; AITR), a co-stimulatory immune checkpoint protein in the TNF superfamily, plays a crucial role in immune regulation. It is widely expressed on DCs, macrophages, T cells, B cells, and endothelial cells (175–177). Its ligand, GITRL (GITRL; AITRL; TL6), is expressed on antigen-presenting cells and vascular endothelium (178, 179). GITR significantly impacts T cell subset activation, proliferation, and polarization, and can activate mature B cells to induce antibody production (180, 181). GITRL expression on B cells is essential for driving Treg proliferation (181). Notably, Foxp3^+^ natural Tregs highly express GITR, and mice lacking these cells suffer severe multi-organ inflammation, including fatal autoimmune myocarditis (182). GITR stimulation in monocytes/macrophages activates NF-κB-dependent MMP-9 and TNF-α production. In atherosclerotic plaques, GITR’s staining pattern overlaps with that of MMP-9 and TNF-α, suggesting GITR-mediated macrophage activation may promote atherosclerosis development and plaque instability (175). This activation also increases the release of cytokines like ICAM-1, TNF-α, IL-6, and IL-8 (183). Endothelial cells, through GITRL, bind to GITR on immune cells, upregulate ICAM-1 and VCAM-1, thereby promoting leukocyte infiltration (184).

During the pathological progression of atherosclerosis, the GITR/GITRL system demonstrates a pronounced pro-inflammatory tendency. Clinical evidence indicates that elevated GITR expression in human carotid plaques is associated with vulnerable plaque phenotypes, cerebrovascular event risk, and elevated levels of local pro-inflammatory cytokines/chemokines (e.g., IL-6, CCL-2, CCL4, CCL5) and matrix metalloproteinases (MMP-1, MMP-9) in conjunction with tissue inhibitor of metalloproteinase-1 (TIMP-1) (185). Animal models further substantiate the pathological role of this pathway. *GITR* ^-/-^ *ApoE* ^-/-^ mice demonstrate significantly diminished plaque areas, characterized by reduced macrophage infiltration, smaller necrotic cores, and thicker fibrous caps, indicative of plaque stabilization (185). Furthermore, monocytes from these mice display reduced integrin levels, impaired endothelial recruitment, and diminished ROS production, while macrophages exhibit attenuated cytokine secretion and migration capacity (185). Targeted inhibition of TNFSF18 (through specific inhibitors or si-TNFSF18 plasmids) significantly diminishes coronary microthrombosis and plaque burden (186). Mechanistically, this effect results from two interconnected processes: firstly, the suppression of pro-inflammatory cytokines such as TNF-α, TNF-β, and IL-1β; and secondly, the inhibition of STAT1 phosphorylation, which subsequently leads to the downregulation of adhesion molecules including VCAM-1 and ICAM-1, as well as integrin subunits integrin subunit alpha D and integrin subunit beta 3. These dual actions collectively produce synergistic anti-inflammatory and anti-adhesive effects (186). In contrast to these findings, Meiler et al. (187) reported that B cell-specific GITRL overexpression provides atheroprotective effects in *Ldlr* ^-/-^ mice, associated with elevated CD4^+^ effector memory T cells, Treg cell populations, and IL-2 production (187). This discrepancy may originate from the cell-specific focus (B cells versus pan-immune cell targeting in *GITR* ^-/-^ and inhibitor models). The pro-inflammatory nature of this pathway is further substantiated in other vascular models: GITR deficiency diminishes P/E-selectin and ICAM-1 expression in ischemia-reperfusion injury, whereas GITR agonist treatment intensifies post-stroke inflammation and neural stem cell apoptosis (188).

Presently, investigational agents targeting the GITR remain limited in scope and progression within global drug development, with clinical research in this domain remaining scarce. Most candidates are confined to early-phase clinical trials (Phase I/II), and no approved therapies or antagonistic agents exist to date—all active modalities predominantly focus on agonist-based strategies. Considering the elevated GITR expression detected in atherosclerosis patients, coupled with the protective effects of GITR blockade against AS progression, GITR stands out as a promising diagnostic biomarker and therapeutic target for future research.

### 4.5 CD70-CD27

CD27 (also known as TNFRSF7), a type I transmembrane glycoprotein, is physiologically expressed on CD4^+^ and CD8^+^ T cells, NK cells, and thymocytes, and is induced during primary immune activation of B cells (189). CD70, the sole ligand for CD27, demonstrates tightly regulated and transient expression exclusively on antigen-activated B and T cells, NK cells, and mature dendritic cells (190). Upon CD27 activation by CD70, the extracellular domain of CD27 is cleaved and released into circulation as a soluble fragment (sCD27) (191). The CD70-CD27 interaction stimulates CD4^+^/CD8^+^ T-cell proliferation, cytokine production, and cytotoxic T-cell response development, while preventing developing thymic Treg cells from apoptosis and increasing Treg frequency (192). Furthermore, it enhances immunoglobulin production by promoting plasma cell differentiation (192). Notably, CD27 expression on Tregs identifies a Treg subset (CD27^+^ Tregs) with potent immunosuppressive capacity, whereas the CD27^−^ Treg subset exhibits moderate inhibitory activity (193).

The expression of CD27 and CD70 is significantly correlated with the initiation and progression of ASCVD. Proteomic analyses demonstrate that elevated plasma levels of CD27 protein show a significant causal relationship with CAD (194). Increased susceptibility to large-artery atherosclerotic stroke is closely correlated with enhanced CD27 expression on memory B cells, IgD^−^CD38^+^ cells, and unswitched memory B cells (195, 196). Furthermore, CD70 expression is significantly elevated in ruptured carotid plaques compared to stable plaques (197). Following AMI, circulating sCD27 levels exhibit a pronounced upward trend (198). In patients with ST-segment elevation myocardial infarction (STEMI), both CD27^+^ Treg and CD27^−^ Treg populations are diminished, with a relative shift toward the CD27^−^ Treg subset (199).

The CD27-CD70 signaling axis exerts multidimensional regulatory effects in the pathogenesis of atherosclerosis. Mechanistically, this pathway participates in the initial disease phase by modulating endothelial NO metabolism and redox homeostasis. CD70 gene silencing significantly reduces the expression and activity of endothelial nitric oxide synthase (eNOS), leading to diminished NO bioavailability and impaired endothelial repair capacity, alongside upregulated NADPH oxidase complex proteins and elevated ROS levels (200). Conversely, CD70 overexpression not only enhances eNOS activity to boost NO levels but also counteracts TNFα-induced suppression of eNOS mRNA (200). During plaque formation, immune cell recruitment triggers reprogramming of endothelial precursor mRNA splicing patterns, which suppresses immune responses through CD27 upregulation (201). Notably, this pathway also plays a critical role in post-ischemic repair. CD70-deficient mice in hindlimb ischemia models exhibit reduced collateral circulation, impaired angiogenesis, and delayed blood flow recovery (199). Furthermore, CD70 antibody treatment in MI models amplifies pro-inflammatory cytokines (e.g., TNF-α, IL-1β, IL-6), reduces Treg infiltration and IL-10 expression, inhibits type I/III collagen synthesis (thereby impairing extracellular matrix remodeling), and exacerbates tissue damage via neutrophil-mediated MMP-9 elevation (198).

The CD70-CD27 signaling axis exerts complex effects in atherosclerosis by regulating diverse immune cells, with its net effect depending on the dynamic balance between cellular subsets. In *ApoE* ^−/−^ mice with myeloid-specific CD70 deficiency, plaque necrotic core volume expands, and macrophage infiltration intensifies (197). This is closely related to downregulated macrophage SR expression, causing impaired oxLDL uptake and reduced ATP-binding cassette transporters, leading to obstructed cholesterol efflux. This may potentially be attributable to enhanced sensitivity of Ly6Chi monocytes to apoptotic signals (202). At the Treg regulatory level, CD27 co-stimulatory signal deficiency in *CD27* ^-/-^ *ApoE* ^-/-^ mice aggravates plaque burden and local inflammation. In contrast, adoptive transfer of *CD27* ^+/+^ *ApoE* ^-/-^ Tregs reverses these pathological changes, confirming that this axis suppresses AS progression by maintaining Treg numbers and immunosuppressive function (203).

Unlike conventional costimulatory molecules, CD27/CD70 activates T cells while providing significant protection against atherosclerosis. The protective mechanisms likely involve multi-target regulation, including maintaining vascular homeostasis by enhancing endothelial NO synthesis, augmenting Tregs’ immunosuppressive functions, and modulating macrophage lipid handling to reduce lipid accumulation. CTX130, a CRISPR-Cas9-engineered allogeneic anti-CD70 CAR T-cell therapy, demonstrates promising efficacy in advanced clear cell renal cell carcinoma, including the first reported complete response in renal cell carcinoma (204). It also exhibits manageable safety and robust objective response rates in relapsed/refractory T-cell malignancies (205). Building on this, the next-generation CAR T-cell therapy CTX131, which incorporates potency-enhancing gene edits, is undergoing clinical development. Future studies could evaluate CD70-targeted therapies in atherosclerosis, extending beyond oncology. Despite limited clinical translational studies directly linking CD27/CD70 to cardiovascular disease outcomes, its unique pathological regulatory properties make it a promising candidate for precision immunotherapy targeting AS.

### 4.6 CD30L-CD30

CD30 (TNFRSF8), a transmembrane receptor of the TNF superfamily, is expressed on activated T cells, B cells, NK cells, and lymphoid precursor cells (206). Its ligand CD30L (TNFSF8/CD153) is detectable on resting/activated B cells, activated T cells, NK cells, eosinophils, monocytes, and mast cells (207). The CD30-CD30L interaction activates downstream NF-κB signaling pathways, regulating cellular proliferation, differentiation, and apoptosis (207). Notably, CD30L also mediates reverse signaling in antigen-presenting cells, establishing a bidirectional communication mechanism critical for immune homeostasis (208).

Emerging evidence implicates the CD30-CD30L axis in atherosclerotic plaque destabilization. Activated CD30^+^ cells predominantly localize to the superficial regions of rupture-prone plaques, particularly within inflammatory infiltrates adjacent to rupture sites. This suggests their involvement in plaque-associated inflammatory cascades. CD30 expression correlates positively with plaque rupture frequency and severity. Activated CD30^+^ cells are found in AS plaque rupture sites, mainly in superficial regions and inflammatory infiltrates near ruptures, indicating CD30’s close link to inflammatory responses at these sites (209). The spatial association of CD30^+^ cells with mural thrombi further implies their potential role in thrombotic events (210). Mechanistically, FOKS et al. (211)demonstrated that anti-CD30L antibody treatment reduced aortic root atherosclerotic lesions by 35% in *Ldlr* ^-/-^ mice, independent of plasma cholesterol levels or lesional macrophage/collagen content. This finding underscores the pathway’s specific immunomodulatory function through T cell response inhibition. The pathological relevance is further corroborated by elevated CD30L expression in cardiomyocytes of acute myocarditis patients, highlighting its broad involvement in cardiovascular inflammatory pathologies (212).

Currently, CD30-targeted therapies are mainly applied in hematologic malignancies. Brentuximab vedotin (BV), an antibody-drug conjugate (ADC), has achieved breakthrough results in CD30-positive lymphoma by precisely delivering microtubule inhibitors. Its success in hematologic tumors inspires exploration of CD30 targeting in other diseases. However, data mining analysis of adverse drug events from the JADER database revealed significant cardiotoxicity risk signals associated with BV, primarily including severe adverse events such as left ventricular dysfunction and cardiomegaly (213). These findings highlight the importance of monitoring cardiovascular-related events during BV therapy. A Phase I clinical trial (NCT05603182) evaluating the safety, tolerability, and pharmacokinetics of PRA052, an investigational anti-CD30L monoclonal antibody antagonist, in healthy volunteers has been completed, but no results have been publicly disclosed. Given the low CD30 expression in healthy tissues and its specific expression in atherosclerotic plaques, CD30 shows potential as a biomarker, yet further research is needed to confirm this (214).

### 4.7 TL1A-DR3

The DR3 (also known as TNFRSF25), a type I transmembrane protein of the TNF receptor superfamily, pairs with tumor necrosis factor-like cytokine 1A (TL1A; VEGI; TNFSF15), the sole confirmed ligand of DR3 to date, which exists as a single-pass type II transmembrane protein (215). DR3 expression is observed in lymphocytes, NK cells, endothelial cells, and macrophages, while TL1A is primarily expressed by umbilical vein endothelial cells, monocytes, macrophages, and dendritic cells (216–218). Although the DR3-TL1A pathway exhibits dual pro-inflammatory and pro-apoptotic properties, recent investigations have revealed its predominant activation of MAPK, NF-κB, and PI3K signaling cascades driving pro-inflammatory responses (217, 218). The DR3-TL1A interaction promotes atherogenesis by inducing pro-atherogenic cytokines and chemokines such as TNF-α, MCP-1, IL-8, and MMP-9 (219). Mechanistically, TL1A upregulates cholesterol uptake-associated genes, including Scavenger Receptor Class A, CD36, and lipoprotein lipase, while downregulating cholesterol efflux-related genes such as ATP-binding cassette transporter A1 (ABCA1), ATP-binding cassette transporter G1 (ABCG1), and apoE. This action drives macrophage foam cell formation (220). Synergistic effects between TL1A and IL-12/IL-18 enhance IFN-γ production in human peripheral blood T cells and NK cells (221), while combined TL1A and IFN-γ signaling amplifies MMP-9 generation (222). Furthermore, TL1A synergizes with IL-17A to induce a disintegrin and metalloproteinase with thrombospondin motifs (ADAMTS) protease expression, which critically contributes to atherosclerotic plaque pathogenesis. Collectively, the DR3-TL1A axis exacerbates atherosclerotic plaque formation and instability through coordinated pro-inflammatory activation and disruption of cholesterol homeostasis.

Numerous studies have demonstrated a significant association between DR3 and TL1A expression and cardiovascular diseases. Elevated TL1A levels have been detected in the peripheral blood of patients with various vascular pathologies, including unprovoked venous thromboembolism, AMI, and CAD (223–226). Notably, TL1A concentrations in peripheral blood showed positive correlations with disease severity in patients with AMI and CAD. DR3 expression on peripheral blood mononuclear cells was found to be markedly upregulated following TNF-α stimulation, lipopolysaccharide activation, or differentiation into macrophage-like cells (227). Kang et al. (219)first provided direct evidence of TL1A and DR3 involvement in atherogenesis through their identification of high-level co-expression in foam cell-rich regions of human carotid atherosclerotic plaques. Current research has identified TNFRSF25 (the gene encoding DR3) as a critical diagnostic gene for atherosclerosis, highlighting its potential as a novel biomarker for this pathology (228).

The DR3-TL1A pathway is tightly regulated by multiple inhibitory mechanisms *in vivo*. Competitive binding between DcR3 and DR3 exerts anti-inflammatory effects (229). Mouse hindlimb ischemia studies revealed that bk-c-kit^+^ cells promote therapeutic angiogenesis through exosomal miR-3059-5p-mediated TL1A inhibition (230). TL1A can be cleaved from the plasma membrane as a soluble form via metalloproteinase activity in endothelial and dendritic cells (231). This suggests that inhibiting metalloproteinases to reduce TL1A cleavage and release may alleviate inflammatory responses. Notably, Zhao et al. (232)demonstrated in *ApoE* ^-/-^ mouse models that while TL1A promotes macrophage-derived foam cell formation by inducing CD36 expression and suppressing ABCA1/G1 expression, it concurrently enhances ABCA1/G1 expression and liver X receptor α/β activation to facilitate cholesterol efflux. This dual mechanism reduces VSMC-derived foam cell formation, ultimately significantly decreasing atherosclerotic lesion areas in the aorta and aortic root while enhancing plaque stability. This paradoxical phenomenon may arise because foam cells in atherosclerotic lesions of *ApoE* ^-/-^ mice predominantly originate from VSMCs, whereas SMCs constitute at least 50% of foam cells in human atherosclerosis (233). Both TL1A-targeted therapies and TL1A inhibition hold clinical relevance, warranting context-specific investigations.

The DR3-TL1A interaction demonstrates significant cardiovascular implications. Current research targeting the TL1A pathway remains limited, with no approved therapies. Investigational agents (e.g., Tulisokibart, PF-06480605, and TEV-48574) primarily focus on inhibiting TL1A for Crohn’s disease (CD) and ulcerative colitis (UC). Tulisokibart and PF-06480605 are currently recruiting participants in Phase III trials for both UC and CD. Recent findings demonstrate promising efficacy signals: in a Phase 2a induction trial, the anti-TL1A monoclonal antibody Tulisokibart showed potential efficacy and favorable tolerability in patients with moderate-to-severe CD (234). Similarly, a Phase 2a single-arm study of anti-TL1A antibody PF-06480605 revealed statistically significant endoscopic improvement and an acceptable safety profile in patients with moderate-to-severe UC (235). Given the elevated TL1A levels in the peripheral blood and plaque phenotypes of atherosclerosis patients, along with its dual effects on macrophage-derived and SMC-derived foam cells, TL1A emerges as a promising biomarker and therapeutic target. Future studies should focus on its cell-type-specific mechanisms and translational potential in cardiovascular pathologies.

### 4.8 LIGHT/LTα_1_β_2_-HVEM/LTβR

Lymphotoxin-alpha (LTα; TNFSF1; TNFβ) and lymphotoxin-beta (LTβ; TNFSF3) are two cytokines of TNFSF. LTα can form a cell surface-bound heterotrimer, LTα_1_β_2_, by associating with LTβ (236, 237). This type II transmembrane protein exclusively binds to the lymphotoxin-beta receptor (LTβR; TNFRSF3) (238). The LTα_1_β_2_ complex is predominantly expressed by lymphocytes, including activated T cells, B cells, and NK cells, but is absent on monocytes and macrophages. In contrast, LTβR is primarily expressed by stromal cells such as endothelial and epithelial cells, but is not detected on T or B lymphocytes, primary monocytes, or peripheral dendritic cells (239, 240). This complementary expression pattern highlights the LTα_1_β_2_-LTβR interaction as a critical molecular bridge. It facilitates cross-talk between lymphoid and non-lymphoid cellular compartments and coordinates immune-stromal interactions in specialized microenvironments.

The binding of LTα_1_β_2_ to LTβR activates intracellular signaling pathways, such as NF-κB, JNK, and p38 MAPK (236). This occurs through the recruitment of adaptor molecules like TRAF3, thereby regulating cellular functions and playing a pivotal role in lymphoid tissue development (241). In SMCs and ECs, LTβR signaling triggers both the canonical and non-canonical NF-κB pathways. This activation induces the expression of inflammatory cytokines, chemokines, and adhesion molecules that mediate immune cell recruitment, including CXCL1, CXCL5, CXCL8, GM-CSF, CCL2, CCL5, CCL20, ICAM-1, VCAM-1, and E-selectin (240). These factors induce endothelial inflammation and promote monocyte migration through LTβR-mediated release of CCL5 and TNF-α from monocytes (242–245). This contributes to macrophage-driven inflammation in atherosclerotic lesions. SMCs located beneath the intimal plaque are stimulated via LTβR signaling to express CXCL13 and CCL21, driving the recruitment of T/B-cell aggregates and facilitating the formation of aortic tertiary lymphoid organs. This process mediates the propagation of adventitial inflammation into the intima. In murine solid fibrosarcoma models, LTα_1_β_2_-LTβR signaling enhances macrophage inflammatory protein-2 production, promoting tumor angiogenesis (246). However, the role of LTβR in atherosclerosis remains debated. In *TNFR1* ^-/-^ *TNFR2* ^-/-^ mice, LTβR signaling in macrophages upregulates ABCA1 protein expression, enhancing cholesterol efflux and potentially exerting protective effects in early atherosclerotic lesions (247).

Circulating LTβR levels exhibit significant correlations with coronary artery calcium, aortic plaque burden and aortic wall thickness (248). In other cardiovascular diseases, such as heart failure and MI, elevated LTβR expression on endothelial cells has also been observed (249, 250). Multiple studies associate LTα alleles with cardiovascular risk factors (249–251). Although the precise role of LTβR in atherosclerosis remains incompletely understood, pharmacological inhibition of LTβR significantly reduces aortic plaque burden and macrophage infiltration in atherosclerotic lesions (252). Liang et al. (253) demonstrated that treatment with paeonol in C57BL/6J mice significantly attenuates atherosclerotic progression and stabilizes vulnerable plaques in *ApoE* ^-/-^ mice. This protective effect occurs through suppression of the LTα1β2-induced LTβR/NIK/caspase-3 signaling pathway, which mediates VSMC apoptosis *in vitro*. These findings collectively suggest that targeting LTβR signaling remains a promising therapeutic strategy for atherosclerosis management.

The Herpes Virus Entry Mediator (HVEM; TNFRSF14) interacts with several TNFSF ligands, including lymphotoxin-like inducible protein that competes with glycoprotein D for herpesvirus entry mediator (LIGHT; TNFSF14), LTα, B- and T-lymphocyte attenuator (BTLA), and CD160. LIGHT, a type II transmembrane protein produced by activated T cells, monocytes, granulocytes, and immature dendritic cells, is also released by activated platelets (254, 255). The soluble homotrimeric LTα3 isoform competitively binds HVEM, demonstrating cross-reactivity between the TNFRSF14/TNFSF14 and LTβR/LTα systems (256). HVEM is prominently expressed on lymphocytes and peripheral blood leukocytes, including CD4^+^ and CD8^+^ T cells, CD19^+^ B cells, and monocytes (257, 258). LIGHT-HVEM binding activates multiple signaling pathways, including MAPKs, PI3K/Akt, NF-κB, JNK, Src family kinases, and focal adhesion kinase, while downregulating p21, p27, and p53. This signaling cascade reciprocally upregulates cyclin D and retinoblastoma protein hyperphosphorylation, inducing chemokine secretion of interleukin-8 and growth-regulated oncogene-alpha, surface expression of adhesion molecules ICAM-1 and VCAM-1, release of prostaglandin I2, and upregulation of cyclooxygenase-2 (259, 260). In endothelial cells and macrophages, LIGHT-HVEM signaling through JNK pathways enhances protease-activated receptor-2 expression, potentiating IL-8 and MCP-1 release (261). Synergistic interactions with IFN-γ markedly induce TNF-α/IL-8 secretion and MMP-9, MMP-1, and MMP-13 production, alongside expression of TIMP-1 and TIMP-2, thereby promoting macrophage migration and VSMC proliferation (222). Furthermore, LIGHT activates NF-κB signaling to suppress lipolytic gene expression while enhancing lipogenic gene transcription and oxLDL-induced inflammatory responses in the Tibetan Human Peripheral blood monocytic cell line macrophages (262). This exacerbates hypertriglyceridemia and hypercholesterolemia, which are key pathogenic mechanisms in atherosclerosis (263).

Both LIGHT and HVEM exhibit elevated expression in atherosclerotic lesions. In patients with stable CAD after percutaneous coronary intervention, elevated soluble TNFSF14 serves as an independent predictor of cardiovascular events and significantly augments the prognostic prediction value of high-sensitivity C-reactive protein (264). LIGHT is predominantly localized to macrophage-derived foam cell-rich regions, and increased LIGHT expression has also been detected in other cardiovascular pathologies, including heart failure and AMI (249, 258, 265). HVEM binding to CD160 triggers rapid phosphorylation of ERK1/2 and AKT, enhancing NK cell cytotoxicity, while BTLA counteracts CD160 activation through competitive HVEM binding (266). The upregulated CD160 expression observed in atherosclerotic patients correlates with pro-inflammatory states, suggesting BTLA as a potential therapeutic target (267). Additionally, Heo et al. (268) demonstrated that emodin and rhein reduce LIGHT-induced ROS production and NADPH oxidase p47 activation, subsequently decreasing phosphorylation of p38 MAPK and IκB-α. This mechanism downregulates CCR1, CCR2, and ICAM-1 expression while suppressing IL-8, MCP-1, TNF-α, and IL-6 production. Kali et al. (258) further proposed that aspirin may confer therapeutic benefits in atherosclerosis by inhibiting platelet-derived LIGHT release.

Current therapeutic strategies targeting the LIGHT/LTα_1_β_2_-HVEM/LTβR pathways remain predominantly in preclinical development. Antagonist drugs have shown markedly sparse clinical advancement in recent years, and no approved pharmaceuticals exist to date. However, given their pro-inflammatory roles in atherosclerosis and disease-associated upregulation, LIGHT, HVEM, and LTβR represent promising candidates for future development as both diagnostic biomarkers and therapeutic targets.

### 4.9 BAFF/APRIL–BAFFR/BCMA/TACI

B cell-activating factor (BAFF; TNFSF13B; BLyS) and a proliferation-inducing ligand (APRIL; TNFSF13A) are TNF superfamily ligands expressed as transmembrane proteins or soluble cytokines. BAFF exerts its functions via three receptors: BAFF-receptor (BAFF-R; TNFRSF13C), B-cell maturation antigen (BCMA; TNFRSF17), and transmembrane activator and calcium modulator and cyclophilin ligand interactor (TACI; TNFRSF13B). In contrast, APRIL binds only to BCMA and TACI, not BAFF-R. In healthy individuals, BAFF and APRIL are primarily produced by myeloid cells (e.g., dendritic cells, monocytes/macrophages, neutrophils) and are widely distributed across tissues, while their receptor expression is largely restricted to specific immune cells (269, 270). Although the BAFFR/BCMA/TACI–BAFF/APRIL axis does not function as classic co-stimulatory molecules, BAFF and APRIL critically regulate B-cell differentiation, proliferation, survival, and functional responses (e.g., humoral immunity, antibody production), with complementary roles between the two ligands (270). Furthermore, beyond B cells, BAFF and APRIL modulate diverse immune cells, including T cells, monocytes, dendritic cells, NK cells, megakaryocytes, and platelets, thereby influencing the pathogenesis of inflammatory diseases (270).

Elevated expression of BAFF and APRIL has been consistently detected in human atherosclerotic plaques. APRIL is predominantly localized to the basement membrane and endothelial cell surfaces of the intimal layer, accounting for approximately 0.5% of total plaque protein content (271–273). Circulating BAFF levels are significantly increased in patients with CAD and AMI. Notably, BAFF demonstrates diagnostic utility, achieving 75.0% sensitivity and 71.4% specificity in identifying CAD patients with high SYNTAX scores, and 75.5% sensitivity with 72.8% specificity for stratifying AMI patients with elevated GRACE risk scores (274). Furthermore, elevated BAFF levels during the acute phase independently predict the incidence of major adverse cardiovascular events in STEMI patients, underscoring its prognostic value (275, 276).

BAFF appears to mediate atheroprotective effects. Tsiantoulas et al. (271)demonstrated that in *ApoE* ^-/-^ and *Ldlr* ^-/-^ mice, treatment with BAFF-neutralizing antibodies induced features of advanced atherosclerosis, including elevated levels of pro-inflammatory factors KC and MCP-1. However, plaque size remained unchanged in *Ldlr* ^-/-^ mice, potentially reflecting stage-specific roles of BAFF in atherosclerosis. In *ApoE* ^-/-^ mice, BAFF neutralization significantly reduced B2 cell populations and antibody levels, yet paradoxically exacerbated plaque burden with increased necrotic core formation and decreased collagen deposition. This paradoxical effect was mechanistically linked to the repression of macrophage IRF7-dependent TLR9 responses, including suppression of proatherogenic CXCL10 production. Conversely, Jackson et al. (277) reported that myeloid-specific BAFF overexpression in *BAFF-Tg*/*ApoE* ^-/-^ mice generated atheroprotective IgM antibodies targeting phosphorylcholine and malondialdehyde-modified LDL. Despite concurrent increases in pro-inflammatory IgG subclasses (IgG2b and IgG2c), aortic root atherosclerotic plaques were reduced by approximately 80%. Subsequent experiments have confirmed that the atheroprotective effects of BAFF are B cell-dependent, involve antibody production, and are mediated by TACI, underscoring its pathway-specific therapeutic potential. These studies collectively highlight the intricate and multifaceted role of BAFF in atherosclerosis, suggesting that its effects are highly dependent on the specific cellular and molecular contexts in which it operates.

In contrast to the atheroprotective effects mediated by TACI, BAFF-R—another receptor for BAFF–exerts proatherogenic activity in a B-cell-dependent manner. *BAFF-R* ^-/-^ *ApoE* ^-/-^ mice exhibited significant reductions in aortic atherosclerotic lesions (23). BAFF-R deficiency markedly decreased B2 cell populations while preserving B1a cells, alongside reduced IgG and IgM deposition in plaques and plasma. Additionally, expression levels of TNF-α, IL-1β, MCP-1, and VCAM-1 were downregulated. This was accompanied by diminished infiltration of macrophages, dendritic cells, CD4^+^ T cells, and CD8^+^ T cells in lesions, as well as decreased numbers of proliferating cell nuclear antigen-positive proliferating cells. Consistent findings were observed in another study: *BAFF-R* ^-/-^ *ApoE* ^-/-^ mice showed significantly reduced anti-oxLDL IgG/IgM antibodies, depletion of major B2 cell subsets (follicular and marginal zone B cells), and smaller plaque volumes (278). However, adoptive transfer of B cells reversed the atheroprotective effects caused by B-cell deficiency. Similarly, *BAFF-R* ^-/-^ *Ldlr* ^-/-^ mice displayed reduced plaque size and T-cell infiltration without alterations in dendritic cell activation (279).

Bernelot et al. (280) demonstrated in *ApoE* ^-/-^ *APRIL-Tg* mice that APRIL overexpression did not significantly alter atherosclerotic plaque volume or necrotic core area but induced features of plaque stability, including increased SMC content. Elevated IgM levels in plasma and plaques were linked to expanded B1a lymphocyte populations, while serum IgG levels increased despite stable total B-cell counts. Tsiantoulas et al. (273) further elucidated the atheroprotective mechanism of APRIL, showing that its interaction with heparan sulfate proteoglycan 2 limits LDL retention in the subendothelial space. *Tnfsf13* ^-/-^ *Ldlr* ^-/-^ mice exhibited exacerbated plaque pathology, characterized by enlarged necrotic cores, acellular regions, and higher macrophage content, without B-cell functional defects. Moreover, *Ldlr* ^-/-^ mice with hematopoietic BCMA deficiency showed comparable atherosclerosis severity to controls. Both studies indicate that, unlike BAFF, APRIL’s effects on atherosclerosis are B-cell-independent. Compared to Bernelot et al.’s (280) findings, Tsiantoulas et al. observed more pronounced APRIL-mediated protection, likely due to localized effects of APRIL within the arterial wall. This is supported by the fact that T-cell-specific human APRIL overexpression in *ApoE* ^-/-^ mice failed to alter plaque size. Furthermore, a non-canonical APRIL was identified, and its association with long-term cardiovascular mortality in atherosclerosis patients remained independent of traditional risk factors.

The impact of BAFF on atherosclerosis appears to be receptor-dependent. BAFF overexpression exerts atheroprotective effects via TACI receptor signaling, while BAFFR receptor inhibition demonstrates protective roles against atherosclerosis in preclinical models. Recent advancements in targeting the BAFF/APRIL system have yielded clinically approved therapies: belimumab (BAFF inhibitor) and telitacicept (TACI-Fc fusion protein) for SLE, offering novel therapeutic avenues. Concurrently, BCMA-targeted (e.g., elranatamab) therapies–including CAR-T cell therapy, bispecific antibodies, and ADCs—have revolutionized treatment for hematologic malignancies such as multiple myeloma. Clinical trials further explore BAFF/APRIL axis modulation for autoimmune and hematologic disorders. Notably, belimumab-treated SLE patients exhibit enhanced high-density lipoprotein cholesterol (HDL-C) efflux capacity and restored antioxidant function, alongside favorable HDL lipidomic profiles comparable to healthy controls. Longitudinal observational studies confirm sustained HDL level elevation post-belimumab therapy, underscoring its potential for ASCVD risk mitigation (281, 282).

Targeted drug development against the BAFF/APRIL system is advancing toward diversification and depth, offering promising solutions for multiple diseases. However, the precise mechanisms and interaction networks of BAFF and APRIL with their receptors in AS pathogenesis require further elucidation. Future studies should prioritize monitoring the effects of belimumab and telitacicept on AS progression in SLE patients, leveraging insights from their established immunomodulatory and lipid-regulating properties.

### 4.10 Other members of the TNF superfamily

The ligand-receptor interactions within the TNF superfamily are pivotal for co-stimulatory immune signaling. Other TNF superfamily members also critically regulate immune homeostasis, apoptosis, and inflammation, all of which contribute to atherosclerosis pathogenesis.

TNF-α, a potent pro-inflammatory cytokine primarily secreted by activated monocytes/macrophages, has spurred significant therapeutic advancements. Five TNF-α inhibitors–infliximab, adalimumab, etanercept, golimumab, and certolizumab pegol–are clinically approved, demonstrating robust efficacy in autoimmune diseases like rheumatoid arthritis. This success highlights their potential repurposing for atherosclerosis treatment. This finding also inspires the exploration of TNF-α inhibitors in atherosclerosis, suggesting potential new pathways for prevention and treatment that merit further investigation. The Fas-FasL pathway, a key mediator of apoptotic signaling, plays a crucial role in atherosclerotic pathology. Additionally, TWEAK, TRAIL, and RANKL, along with their receptors, are closely linked to atherosclerosis. They show potential for diagnosis and prognosis assessment, and preclinical studies in mouse models indicate their therapeutic promise. Recent reviews have summarized these findings comprehensively (283–286). As research advances, growing insights into these molecules and pathways mark them as promising new targets for atherosclerosis prevention and treatment. Further probing their specific roles in disease progression will facilitate the development of more targeted treatment strategies for atherosclerosis.

## 5 Future perspectives: the promise and challenges of TNF superfamily in atherosclerosis therapy

In recent years, the successful application of TNF superfamily members in the treatment of autoimmune diseases and tumors has marked a significant breakthrough in medical research. A variety of biologics targeting TNF superfamily members, including CD30, BCMA, TNF-α, RANKL, etc., have gained approval from the U.S. Food and Drug Administration (FDA) and have been successfully applied in clinical practice. Notably, TNF-α and BAFF-R targeted therapies have demonstrated remarkable efficacy in treating autoimmune diseases such as rheumatoid arthritis and systemic lupus erythematosus. This progress not only highlights the therapeutic potential of TNF superfamily members but also paves the way for their application in other disease areas. Recent preclinical evidence suggests that certain TNF superfamily members, such as CD40L and OX40L, play a central role in the progression of atherosclerosis by regulating key pathological processes such as lipid metabolism, plaque formation, fibrous calcification, and plaque rupture. This indicates their great potential as therapeutic targets for ASCVD.

At the mechanistic level, immune checkpoint proteins in the TNFSF family primarily serve as co-stimulatory molecules, but their activation does not uniformly promote AS. For instance, activation of the CD27/CD70 pathway may produce atheroprotective effects, whereas activation of CD40/CD40L, CD137L-CD137, or GITR-GITRL may promote atherosclerosis. Such differences may arise from cell type-specific signaling mechanisms. For example, B cell-specific overexpression of GITRL can induce an atheroprotective phenotype, while systemic knockout of GITR or the use of TNFSF18 inhibitors also shows plaque-reducing and thrombus-inhibiting effects (185–187). In the face of these cell-specific contradictions, it is crucial to develop novel delivery systems that precisely target specific cellular subsets. TRAF-STOPs nanotechnology offers a viable solution by selectively inhibiting CD40-TRAF6 signaling in macrophages, effectively curbing AS progression and avoiding systemic immunosuppression (99). Additionally, statins inhibit inflammatory pathways such as OX40/OX40L and CD40L/CD40, while aspirin reduces circulating sCD40L levels (82–86). These findings provide a solid theoretical basis for combining TNF family inhibitors with traditional anti-AS drugs, such as lipid-lowering and antiplatelet agents.

Beyond the classic co-stimulatory pathways, the BAFFR/BCMA/TACI-BAFF/APRIL axis also significantly impacts AS progression. Notably, targeting BAFFR and TACI may yield opposing effects on AS regulation, underscoring the importance of in-depth signal network analysis. Currently available BAFF/APRIL-targeted therapies, such as belimumab, offer opportunities for exploring their cardiovascular protective effects in clinical settings.

Genetic research further supports the role of TNF superfamily members in AS. Polymorphisms in TNFSF genes are significantly associated with AS susceptibility, and soluble proteins such as sCD40L show a positive correlation with cardiovascular event risk. This suggests a dual potential for TNF superfamily members: as dynamic biomarkers for optimizing cardiovascular risk stratification and as therapeutic targets for developing novel interventions.

## 6 Conclusion

In summary, this paper comprehensively analyzes the important role of the TNF superfamily in AS pathogenesis. Preclinical models confirm that targeting TNF family immune checkpoints can effectively slow AS progression, and other TNF molecules, such as BAFF, APRIL, and TEWAK, also exhibit significant therapeutic potential. Although different receptor targets show varying effects, combining innovative nanodelivery technologies for cell-specific targeting with existing cardiovascular drugs for combination therapy will undoubtedly open up new avenues for ASCVD treatment. However, given that AS is a dynamic pathological process influenced by multiple factors, future research must transcend the limitations of mouse models. It is essential to deeply clarify the spatiotemporal dynamics of TNF signaling networks and construct comprehensive immune regulation maps for each cell type within human systems. This will provide a theoretical foundation for targeting specific downstream signals and cells, ultimately bridging the gap from mechanistic research to clinical translation. As research deepens and technology continues to innovate, the TNF superfamily is expected to become a key target in ASCVD therapy, bringing new hope for the prevention and treatment of cardiovascular diseases.

NF-κB = nuclear factor kappa-β; IκBα = inhibitor of nuclear factor-κB α; NIK = NF-κB-inducing kinase; RIP = receptor-interacting protein; ASK1 = apoptosis signal-regulating kinase 1; AP-1 = activator protein-1; TRAF = TNF receptor-associated factor; ERK= extracellular-signal-regulated kinases; PI3K = phosphatidylinositol-4,5-bisphosphate 3-kinase; AKT = protein kinase B; RelA and RelB = V-rel avian reticuloendotheliosis viral oncogene homolog A and B; RAF = rapidly accelerated fibrosarcoma; MAPKKK = mitogen-activated protein kinase kinase kinase; MKK = mitogen-activated protein kinase kinase; MEK = MAP/ERK kinase; RAS = rat sarcoma protein; mTORC1 = mechanistic target of rapamycin complex 1 ; S1P/YAP = sphingosine 1-phosphate/Yes 1-associated protein.
